# Recent Progress in the Abatement of Hazardous Pollutants Using Photocatalytic TiO_2_-Based Building Materials

**DOI:** 10.3390/nano10091854

**Published:** 2020-09-16

**Authors:** Anantha-Iyengar Gopalan, Jun-Cheol Lee, Gopalan Saianand, Kwang-Pill Lee, Prashant Sonar, Rajarathnam Dharmarajan, Yao-long Hou, Ki-Yong Ann, Venkatramanan Kannan, Wha-Jung Kim

**Affiliations:** 1 Daegyeong Regional Infrastructure Technology Development Center, Kyungpook National University, Daegu 41566, Korea; algopal99@gmail.com (A.-I.G.); kplee@knu.ac.kr (K.-P.L.); 2 Department of Architecture, Seowon University, Cheongju 28674, Korea; uggenius@hanmail.net; 3 Global Centre for Environmental Remediation (GCER), Faculty of Science, The University of Newcastle, Callaghan, New South Wales 2308, Australia; SaiAnand.Gopalan@newcastle.edu.au (G.S.); Raja.Dharmarajan@newcastle.edu.au (R.D.); 4 School of Chemistry and Physics, Queensland University of Technology, 2 George Street, Brisbane, QLD 4001, Australia; Sonar.Prashant@qut.edu.au; 5 Centre for Material Science, Queensland University of Technology, 2 George Street, Brisbane, QLD 4001, Australia; 6 Department of Civil Engineering, Kyungpook National University, 80 Daehakro, Buk-gu, Daegu 41566, Korea; hylmm8988@hotmail.com; 7 Department of Civil and Environmental Engineering, Hanyang University, Ansan 1588, Korea; kann@hanyang.ac.kr; 8 Department of Physics, SCSVMV Deemed University, Kanchipuram 631561, India; kv@kanchiuniv.ac.in

**Keywords:** building materials, photocatalytic, titanium dioxide, pollutant removal, self-cleaning

## Abstract

Titanium dioxide (TiO_2_) has been extensively investigated in interdisciplinary research (such as catalysis, energy, environment, health, etc.) owing to its attractive physico-chemical properties, abundant nature, chemical/environmental stability, low-cost manufacturing, low toxicity, etc. Over time, TiO_2_-incorporated building/construction materials have been utilized for mitigating potential problems related to the environment and human health issues. However, there are challenges with regards to photocatalytic efficiency improvements, lab to industrial scaling up, and commercial product production. Several innovative approaches/strategies have been evolved towards TiO_2_ modification with the focus of improving its photocatalytic efficiency. Taking these aspects into consideration, research has focused on the utilization of many of these advanced TiO_2_ materials towards the development of construction materials such as concrete, mortar, pavements, paints, etc. This topical review focuses explicitly on capturing and highlighting research advancements in the last five years (mainly) (2014–2019) on the utilization of various modified TiO_2_ materials for the development of practical photocatalytic building materials (PBM). We briefly summarize the prospective applications of TiO_2_-based building materials (cement, mortar, concretes, paints, coating, etc.) with relevance to the removal of outdoor/indoor NO_x_ and volatile organic compounds, self-cleaning of the surfaces, etc. As a concluding remark, we outline the challenges and make recommendations for the future outlook of further investigations and developments in this prosperous area.

## 1. Introduction

In recent years, challenges regarding air pollution based on particulate matter (PM) and toxic gases that are arising from anthropogenic or external sources have become severe issues [1]. Research activities are being directed to resolve air pollution-related matters through the process of making practical remedial measures. Air pollution can occur both in indoor (e.g., household) contexts and in outdoor environments. Common hazardous compounds include NO_x_, SO_x_, CO, H_2_S, NH_3_, other nitrogen compounds (e.g., hydrogen cyanide), sulfur-containing compounds (organothiols), hydrocarbons, and a myriad of volatile organic compounds (VOCs) (benzene, toluene, methanol, etc.) which are of significant concern for environmental remediation [2]. The primary sources of these gaseous pollutants are anthropogenic. Mainly, VOCs based on alkanes, aromatics, esters, alkenes, carboxylic acids, and alcohols can exist in indoor environments, especially inside building spaces, originating from adhesives, carpeting, wood products, or cleaning products [3]. These indoor air pollutants can cause health issues, such as breathing problems, for people with compromised or underdeveloped immune systems [4,5]. On the other hand, fossil fuel burning by motor vehicles (including cars, buses, trucks, etc.) and power plants (including coal-fired and natural gas plants) can expel a mixture of pollutants, such as tiny particles or PM, NO_x,_ and VOCs [6,7,8,9]. Besides, secondary pollutants also originate indirectly from the air when primary pollutants react or interact with each other or with other environmentally existing gaseous molecules. For example, chemical interactions between SO_x_ and NO_x_ can produce ground level ozone, and this can happen in the upper atmosphere too, leading to photochemical smog and acid rain [10]. These outdoor and secondary pollutants can cause environmental and human health issues.

Gaseous pollutants are mainly controlled or removed by a few significant techniques: absorption, adsorption, chemical oxidation, and incineration. These techniques may be employed either singly or in combination, which, in turn, can be decided based on the type of pollutant. Gas adsorption is a surface phenomenon where gas molecules are adsorbed or are attracted to the surface and held on the surface of the solid. These approaches for the depollution process, however, are constrained with drawbacks [11]. For example, while activated carbon [12,13] adsorption can lead to secondary pollution, incineration and catalytic oxidation are expensive and consume more energy. Photocatalysis is a rapidly evolving technology for the treatment of air pollutants and is considered as one of the most effective methods to remediate environmental gaseous pollutants. Photocatalysis refers to the conversion of photon energy into chemical energy and the acceleration of photoreactions in the presence of a photocatalyst. Photocatalysis offers several advantages over the conventional catalytic processes that involve time-consuming steps, elevated temperatures, and pressures. Typically, photocatalysis, or photocatalytic oxidation (PCO), has been effectively employed for the removal of low concentrations [i.e., part per billion (ppb) level] of gaseous pollutants, with distinct advantages that include ambient temperature operations, minimum energy consumption, and effectiveness for various contaminants with innocuous final products [14].

Several metal-oxide based semiconductors, such as titanium dioxide (TiO_2_) [15,16,17,18], zinc oxide (ZnO) [19,20,21,22,23,24,25,26,27], copper oxide (CuO) [28,29,30,31], zirconium oxide or zirconia (ZrO_2_), cerium oxide (CeO_2_) [29], tungsten oxide (WO_3_), and tin oxide (SnO_2_), have been successfully demonstrated as efficient photocatalysts for pollutant removal [32,33,34] and utilized in several other applications (energy, environment, and health) [35,36,37]. The semiconductor photocatalytic process includes irradiation of the semiconductor surface with a light having energy greater than the semiconductor’s bandgap. This triggers the excitation of valence band (VB) electrons and transfers to the conduction band (CB) to create electron-hole pair (e^−^-h^+^). These charge carriers subsequently participate in reactions between the pollutant molecules and radicals generated on the surface of the semiconductor [38,39]. The rapid development and convergence of the critical scientific components, such as catalysis science, nanoscience, and material characterizations, form the basis for significant advances in the development of new photocatalysts, photocatalytic reaction mechanisms, and the structure-photo activity relationship between photocatalysts and target pollutants [40,41,42,43,44]. More interestingly, the structural features of photocatalysts can be further fine-tuned to derive enhanced photocatalytic performances for the depollution process [42]. Particularly, semiconductor photocatalysts have been proved to exhibit significant efficiency for the degradation of both organic and inorganic pollutants.

**Scheme 1:**
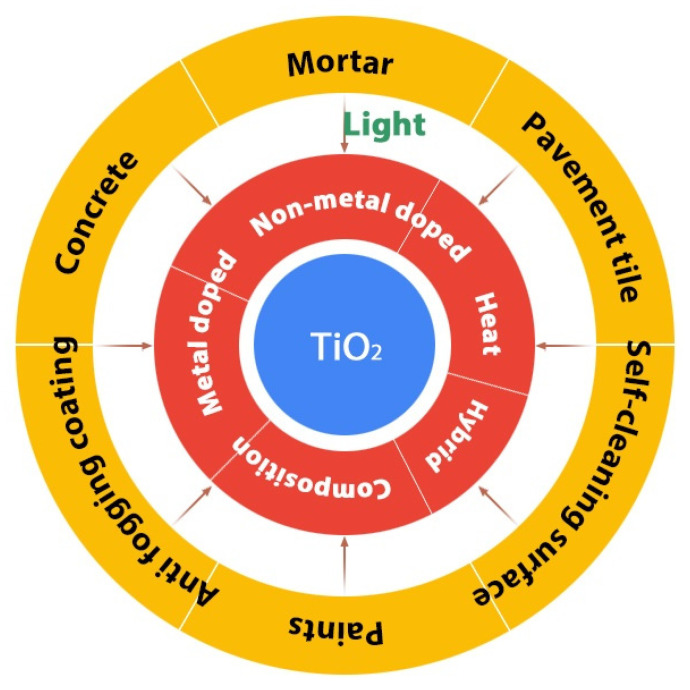
General schematic showing the prospects of titanium dioxide (TiO_2)_ in building-related applications.

TiO_2_ is one of the popular commercial photocatalysts which is prevalently used for the photo-degradation of many pollutants. TiO_2_ offers major advantageous characteristics, such as ease of synthesis and versatility of application. TiO_2_-based photocatalysis has proven to be a very promising advanced oxidation process for the depollution and cleaning of indoor and outdoor air, exhibiting unique advantages over conventional remediation technologies. Through a significant number of research studies, the unique photocatalytic properties of TiO_2_ structures, or the phase compositions that facilitate the oxidation of nitrogen oxide (NO) to nitrogen dioxide (NO_2_) and then to nitric acid (HNO_3_), as well as the conversion of VOCs to CO_2_ and H_2_O under ultraviolet (UV) irradiation, have been well documented. Continuous research activities are being directed towards improving the photocatalytic efficiency of TiO_2_ via preparation of nanostructured TiO_2_, doping with non-metal (C, N, S, and I) and metal ions (Cr, Mn, Fe, and Ni), preparation of different polymorphs and their mixtures, composite formation, etc. (Scheme 1). The commonly known anatase and rutile forms of TiO_2_ belong to the large bandgap semiconductors with bandgap energies of 3.2 and 3.0 eV, respectively [45]. However, chemical/structural modifications of TiO_2_ can result in the decrease in the bandgap in such a way that TiO_2_ could be effectively used for the transformation/degradation of environmental pollutants/contaminants under solar or visible light irradiation.

It is being projected that there will be a three-fold increase in the numbers of commercial and institutional buildings in 2050 as compared to 2010. Globally, buildings account for nearly 20% to 40% of energy consumption in developed countries, even surpassing the possible contributions from the industrial and transportation sectors [46]. As a result, it is believed that the flow of the economy is expected to be largely based on the construction industry. With regards to buildings, construction materials are used in a significant part of their construction, and they are directly exposed to people. It is expected, therefore, that the indoor environment is strongly influenced by the nature of building material and, additionally, by the quality and cost of the construction [47,48]. Extensive academic research activities on various aspects of TiO_2_ significantly provide the basis for the utility of TiO_2_ in practical applications, especially as a photocatalytic construction/building material. The advantages of using TiO_2_ as a photocatalytic building material over the other semiconductor photocatalysts are: (a) less expensive and higher chemical stability and safety; (b) high photocatalytic activity; (c) compatibility with traditional construction materials; (d) effectiveness under solar light irradiation in ambient/atmospheric environments. Based on some laboratory and feasibility studies towards its practical applications [49,50], it has been demonstrated that TiO_2_-based photocatalytically active construction materials can be useful for gaseous depollution and environmental cleaning processes [51]. Also, practical condition test results on the air purification effect of TiO_2_ included cementitious materials that have been reported [52].

In recent years (especially the last five years, 2014–2019), several innovative approaches have been developed towards TiO_2_ modifications, focusing on the improvement of its photocatalytic efficiency. Notably, research has focused on the utilization of many of these advanced TiO_2_ materials and their derived photocatalytic activities for the development of construction materials, such as concrete, mortar, pavements, paints, etc. (Scheme 1). For example, the combination of photocatalytic oxidation activity and photo-induced super-hydrophilicity was utilized to develop TiO_2_-based building materials, such as tiles, coated glasses, paint films, and tent material, having a self-cleaning property with excellent durability. Detailed information has been well demonstrated by researchers based on the results achieved with generalized use of self-cleaning surfaces, which are intended to provide buildings with a long-time stable aesthetic appearance. In parallel, the capability of photocatalysis in cementitious materials has been established in laboratories, at pilot scales, and, more recently, with some specific in-field monitoring programs towards the reduction of the levels of urban pollution and related developments. The additional benefits of improving indoor air quality can be exploited by utilizing photocatalysis involving TiO_2_-inclusive building materials. For example, coating a layer of cement endowed with photocatalytic properties on building walls in conjunction with exposure to appropriate light sources has been demonstrated to decrease pollution levels in confined (indoor) environments. Therefore, indoor or outdoor environments containing low pollutant concentrations (at ppb levels) can be effectively controlled to have minimized pollution levels through the use of photocatalytically active building materials; this forms the basis for a promising field of application. It must be noted that traditional air purification technologies are less efficient and economically less competitive for the treatment of low concentrations of pollutants present in indoor air streams. It is noteworthy that the photocatalytic coating approach is not only useful to retain the aesthetic characteristics of concrete structures based on white cement but also reduces the discoloration effect of the wall surface because of the accumulation of organic pollutants. Even so, adequately designed TiO_2_-cement composites can retain the aesthetic characteristics of building walls over longer durations, even in an aggressive outdoor environment. The significant applications of TiO_2_-based photocatalytic building materials can be grouped under environmental pollution remediation, self-cleaning, and self-disinfecting. However, the inclusion of a TiO_2_ photocatalyst into construction materials, such as cement-based products, or its immobilization onto the external surfaces of other materials, such as construction tiles, has become an appealing challenge for exploiting the unique properties of the PCO process and establishing its practical application for the removal of air pollutants in urban areas.

**Scheme 2:**
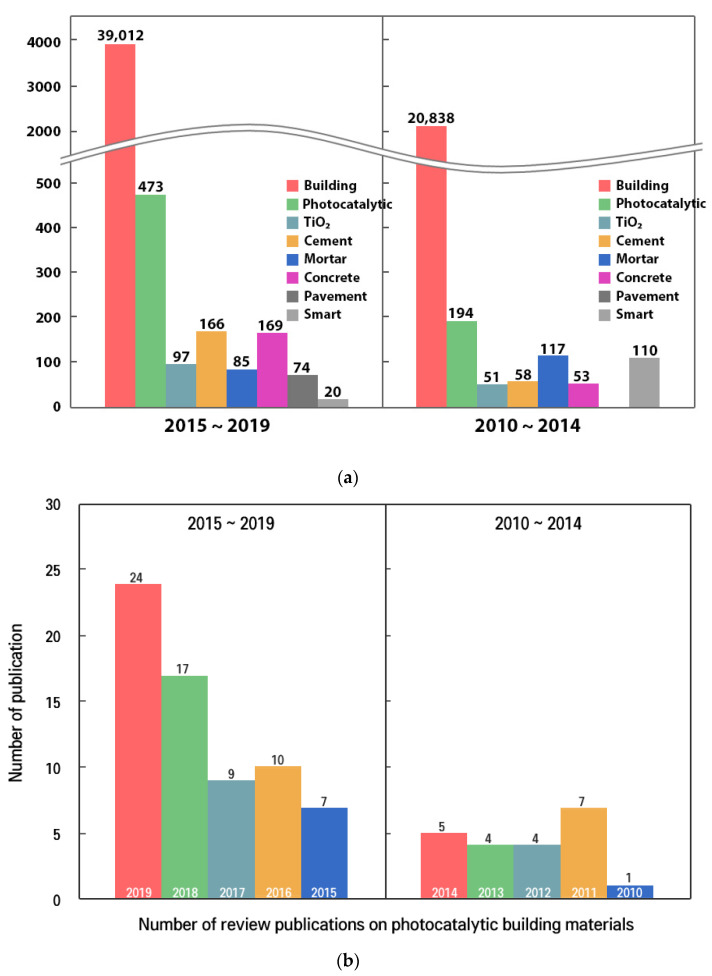
(**a**) Several contributions published annually from 2010 to 2019 on TiO_2_-based photocatalytic materials in various sub-topics and (**b**) Number of published review articles annually from 2010 to 2019 for TiO_2_-based photocatalytic building materials.

On perusal of the published scientific literature through “Web of Science”, using the keyword “building materials” over the years 2010 to 2019, the database indicated the appearance of ~85,140 records (mainly in the form of articles, reviews, and proceedings). Typically, the number of research articles and reviews nearly doubled in the years 2015–2019 as compared to the years 2010–2014 (Scheme 2a). On further refining, for a search with the keyword “photocatalytic”, one could notice that the number of published research articles significantly increased (about 2.5 times) for the years 2014–2019, as compared to the research articles in the years 2010–2014 (Scheme 2a). To note, the number of reviews published on the topic “photocatalytic building materials” also showed such an increasing trend (~21 (2010–2014) vs. 67 (2015–2019) (Scheme 2b). Notably, TiO_2_ (especially the commercial material Degussa-Evonik P25) was predominantly used for the “PBM based applications” against other photoactive materials, indicating the predominance of TiO_2_ over the other photoactive materials. While research activities on “TiO_2_ based building materials” showed a sluggish improvement between the years 2010–2014, a sharp increase in research activities was observed in the years 2015–2019 (Scheme 2b). Also, in recent years, researchers started focusing their interest on developing “smart building materials” using TiO_2_.

**Scheme 3:**
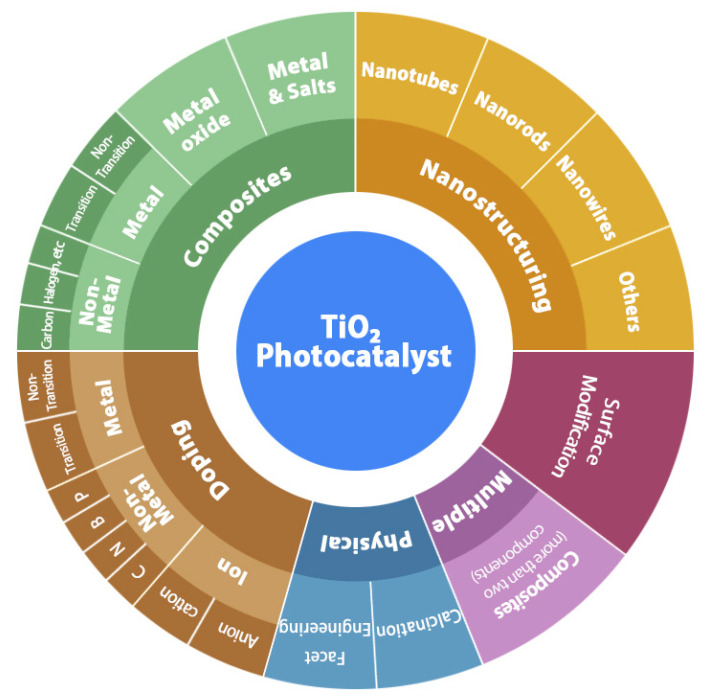
Pictorial representation of the synthetic methodologies and modification strategies of the TiO_2_ photocatalyst.

On close perusal of the literature, we noticed that few review articles had been published in the recent period (2015–2019) on photocatalytic building materials (PBM), focusing on particular topics such as photocatalytic cement-based coatings [53], concrete nanotechnology [54], renewable photocatalysts in concrete [55], nano-inclusions applied in cement-matrices [56], indoor air purification by photocatalysts included concretes [55], etc. These reviews describe only a part of the discussion related to the utilization of TiO_2_ in building/construction materials (Scheme 3). Readers are advised to refer to the review published in 2009 to understand the development of photocatalytic construction and building materials focusing on three major research areas, including air purification, self-cleaning, and self-disinfection [57]. This particular review briefly describes the development of basic research and developments of TiO_2_-based photocatalysis in the field of construction and building materials.

A comprehensive report focusing on the utility of photocatalytic TiO_2_-based building materials, especially in the last few years (2015 to 2019), is highly warranted. Considering the importance of research activities on “TiO_2_ based building materials and related application” and the independent research developments that contributed to improving the photocatalytic properties of TiO_2_, such as enhanced photoactivity, an extension of visible-light photoactivity, etc., this review particularly focuses on the advancements of various kinds of TiO_2_-based building materials, such as cement, mortar, concrete, paints, etc., from 2015 to 2019. The review details the role of this TiO_2_-based building material with relevance to (i) NO_x_ removal, (ii) VOC photo-degradation, and (iii) self-cleaning surface developments, with sub-headings specifying the kind of building material, such as cement, concrete, mortar, and paint. At the end of the review, a summary of the research activities on TiO_2_-based building materials and directions for further research are presented.

## 2. Building Materials

Simple descriptions of some of the essential building materials (BM), such as cement, mortar, and concrete, are presented here for fair comparison among these materials. Lime is primarily comprised of calcium oxide (CaO) and/or calcium hydroxide and/or calcium carbonate (CaCO_3_), which can be obtained from the calcination of limestone (quick-lime) and hydrated lime. In a typical preparation of cement from limestone, a powder mixture comprising of limestone (~70–90%), clays (~5–20%), or alumina, bauxite, etc. (mostly Al_2_O_3_, or Fe_2_O_3_ and SiO_2_), silica (~3–10%), iron ore, or iron oxide (~1–5%) is heated to ~1400 °C in a rotary kiln. During heating, the limestone decomposes to CaO beyond 900 °C, which subsequently, with silica, forms dicalcium silicate 2CaO·SiO_2_ (referred to as C_2_S in the notation of cement chemistry). Further, at temperatures above 1200 °C, the components in the clay, such as Al_2_O_3_ and Fe_2_O_3_, as well as the iron component from iron ore, subsequently react with CaO and form the product tricalcium aluminate, 3CaO·Al_2_O_3_ (by notation as C3A), and calcium aluminoferrite, 4CaO·Al_2_O_3_·Fe_2_O_3_ (by notation as C4AF). Beyond 1250 °C, the ‘aluminate’ minerals that exist in liquid form function as a medium to facilitate the reaction between dicalcium silicate and further CaO to form cement, tricalcium silicate 3CaO·SiO_2_ (C3S). The semi-liquid that is present at about 1300–1400 °C can be further mixed with a cement linker (gypsum, others, etc.) to produce general-purpose cement or Portland cement. The terms cement and concrete are used interchangeably. However, cement is one of the ingredients of concrete. On the other hand, concrete comprises of aggregates, otherwise called gravel (sand and gravel or crushed stone), and paste (a mixture of water and cement). Mortar is another building material derived from cement that is composed of cement, fine sands, lime, and water. TiO_2_ or modified TiO_2_ materials can be included in any of the forms of building materials (cement, concrete, or mortar), and the chemical interactions among the components vary significantly, depending on the method of inclusion of TiO_2_ into these building materials.

Stone is one of the most widely used BMs since ancient times. Long-time exposure of historical constructions, sculptures, and monuments made of stone to weathering conditions and atmospheric pollution can cause a major change in the visual appearance of their surface or blackening of the architectural surfaces. Typically, the surface of the stone is subjected to deterioration over time due to various sources that include interaction with polluting gases and fine particulates. Most of these alterations mainly influence the visual appearance (aesthetics) of the stone itself, besides the loss of the original structural features of the stony material. In particular, research studies revealed that carbonate-based rocks, such as calcarenite and marble, are vulnerable to deterioration due to natural weathering and urban pollution. Also, biotic factors, such as the growth of microorganisms and biofilm formation, can cause degradation of stone surfaces and appearance. These issues can be fully or partially resolved by covering the surfaces of stony architectures with coatings. However, one must remember that a suitable coating for stone should introduce not only effective functions for stone protection, self-cleaning, and preservation of the aesthetic features of the original material, but should also be compatible with previous treatments and ensure stability and durability. It is worthwhile to note that the development of functional coatings with multiple properties, such as weather-resistance, self-cleaning, and a hydrophobic nature, is a promising challenge because of the wide variations in the chemical compositions of stones. A few reports pertinent to the application of TiO_2_ photocatalysis and the related phenomenon of stone coating for preservation are detailed below.

Studies have been focused on the development of functional coatings based on TiO_2_ nanoparticles (NPs) in the process of protecting lapideous (texture of stone) building materials in monuments and artworks of historical and architectural nature [58,59]. The coatings based on TiO_2_ NPs for cultural heritage conservation need to comply with multiple requirements. Functional TiO_2_ NPs based coatings have shown multiple capabilities that include prevention of the bio-deterioration of stony surfaces [60], controlling the formation of black crusts and salts [61], and conferring hydrophobic properties to the surface for reducing the percolation/penetration of water into the pores without affecting the aesthetic appearance [59]. The coating formulation can be made through two different approaches. In the first formulation, TiO_2_ NPs can be incorporated in a commercial polymer host matrix that can be used as a cleanser, protector, or consolidant. The second approach involves the direct application of TiO_2_ NPs onto the stone surface through spray coating. The second approach presents several advantages (using suitable formulations), such as retaining the photocatalytic activity, preserving the aesthetic appearance of the stone surface, and formation of a uniform surface [62]. In this regard, aqueous and alcoholic suspensions of TiO_2_ NPs have been applied to porous and compact limestones [63,64]. TiO_2_-based coatings with NP sizes in the range of 10–20 nm were applied through spray coating, resulting in a uniform coating with photocatalytic activity and the preservation of the stone’s aesthetics [65]. Mesoporous anatase TiO_2_-based coating dispersion in ethylene glycol was applied onto Noto stone and Carrara marble surfaces, and the aesthetic compatibility, capillary absorption coefficient, and photocatalytic activity were reported to be significantly improved [66]. The coating based on TiO_2_ NPs’ dispersion in chloroform was tested on stony materials and the NPs were surface functionalized for improving their dispersibility in the solvent [67]. Other approaches, such as casting and dipping, were attempted for photocatalytic TiO_2_ nanorod-based coatings on Lecce stone surfaces [67]. The spray coating was reported to be cost-effective for obtaining thin and crack-free films which were highly stable on the stone surface [68,69]. Another study has shown that coatings based on TiO_2_ NPs on stones induced a photocatalytic self-cleaning effect on the surfaces [70].

## 3. Integration of Photocatalyst on Building Materials

This heading details the key factors that involve the integration of TiO_2_ photocatalysts with BMs: the preparation of TiO_2_, the BM component to be integrated (e.g., cement, concrete, etc.), and the process steps. Each one of the key factors for the integration of a BM with TiO_2_ contributes to the final photoactivity and performance of the fabricated materials. Firstly, the selection of TiO_2_ NPs with adequate physico-chemical characteristics (size, phase composition, morphology, and surface area) is a core part of their integration into a BM. The process step in the integration can either be mixing or dispersing TiO_2_ into the BM matrix, and this step determines the uniformity and the physico-chemical properties of the TiO_2_ integrated BM. The mixing process can be categorized as either synchronous or latter ad-mixing, depending on the stage at which the mixing of TiO_2_ is performed. However, the mixing process for the integration of TiO_2_ with a BM is constrained with the problem of agglomeration of particles, irrespective of the mixing methodology adopted. Mostly, the smaller sizes and the high surface energy of TiO_2_, as well as the Brownian motion of the smaller particles, can cause particles to agglomerate. The agglomeration of TiO_2_ particles can not only reduce their photoactivity but also their mechanical strength by blocking access to the internal surface of TiO_2_.

Alternatively, the dispersion technique can be a better approach for resolving agglomeration problems. The bead or ball milling technique has been attempted to disperse NPs [71]. However, the problem involved in bead milling is the difficulty of handling the process with slurries having high solid fractions. The ball milling method has advantages, such as low cost, environment-friendly approach, high efficiency and controllability with the parameters, and large scale applicability in industries [72,73]. Ultrasonic irradiation can be another process step for the dispersion of submicrometer sized particles, which can eliminate the agglomeration problem [74,75]. The agglomeration of particles can be prevented by the shock waves generated from ultrasonic irradiation and collapsing cavitations, which causes the agglomerated particles to split due to the collisions. However, ultrasonic dispersion is an expensive technique as it is energy-intensive. To circumvent aggregation problems, the utilization of water-reducing admixtures (plasticizers and superplasticizers) and surfactant treatments have been attempted [76,77]. Surface potential (zeta potential) modulation can be used to prevent the agglomeration of TiO_2_ particles. For example, the zeta potential of cement mortar is the measure of the degree of repelling between particles, and indicates the surface charge states. Zeta potential values lower than −30 mV and higher than +30 mV can be used as guidelines for preventing agglomeration [78]. Importantly, under the condition that the charges of the added TiO_2_ particles and mortars are similar, agglomeration can be minimal, as the particles of each sort are supposed to repel each other. One must note that zeta potential measurements of cementitious materials have generally been done with a low fraction of solids, hence it is challenging to conclude information about the zeta potential values of fresh cement mortars and photocatalytic cement mortars. Recently, spray coating has been tried to create a surface coating on stone or building surfaces [79]. The spray coating formulation was developed based on nitrogen-doped TiO_2_ using a silane coupling reagent, and the coating durability was around 13 months [80] This review mainly focuses on the photocatalytic pollutant removal performance of TiO_2_-loaded BMs and the durability, mechanical strength, and lifetime issues of the integrated materials. There are controversial reports on the durability and mechanical effects of added TiO_2_ into BMs. While it has been reported that nano TiO_2_ is chemically inert in the process of cement hydration, it has also been suggested that it can provide crystal nuclei to promote cement hydration, causing an increase in the compressive strength of mortars [81] Additionally, it has been reported that high brittleness and internal cracks that could be generated due to photocatalyst inclusion are the reasons for the decline in photocatalytic performance and service life of building materials. Readers are advised to refer to the forthcoming sections that deal with uses of TiO_2_-integrated BMs for specific environmental pollutant removal and self cleaning. Also, it should be noted that the PBMs generated through the integration of TiO_2_ with any of the building material constituents should withstand the harsh and aggressive environments in which they are put into use. These requirements are dependent on the strength and durability of the resultant PBMs.

## 4. TiO_2_ Photoactivity under Solar Light

TiO_2_ exists in three different polymorphs: anatase, rutile, and brookite, with a bandgap of 3.2 eV for anatase, ~3.0 eV for rutile, and ~3.2 eV for brookite [82,83]. The bandgap of anatase corresponds to a wavelength of 388 nm, signifying that photo-activation of anatase can happen under irradiation of light with a wavelength lower than 388 nm. Therefore, visible light irradiation is not able to trigger the photocatalytic activity in anatase [84,85,86]. Recently, there is a growing interest in sunlight-driven photocatalytic applications, considering the fact that the UV fraction of the solar spectrum is limited to ~4%; on the other hand, significant proportions of photons can be generated from the visible region (early 50%).

Various strategies have been employed to make TiO_2_ a visible light active material [87]. The techniques/methodologies include surface modification via organic materials, mixing with other visible light active semiconductors, tuning the bandgap by creating oxygen vacancies and doping with metal/nonmetals, and co-doping of nonmetals and metals. Doping elements, such as S, C, N, and B, can create interband energy states between valence and conduction bands, or lower the bandgap and thus extend the visible light absorption capacity of TiO_2_ [88]. The non-metal dopants could substitute the oxygen atoms from the lattice of TiO_2_, create oxygen vacancies, and lower the bandgap. Similarly, noble metals and other semiconductors can form heterojunctions with TiO_2_ and enhance the photocatalytic activities of TiO_2_ [86,89,90,91]. Readers are advised to refer to the published reviews on the mechanism of visible light activity induction and the methodology of modification of TiO_2_ for achieving visible light activities [92,93,94,95,96,97]. It must be noted that, keeping in mind the fact that sunlight is the natural source for an enormous proportion of visible light, research is focused on harnessing sunlight to meet the urgent demand for a cleaner environment. Architects and structural engineers are directing their attention towards the development of advanced photocatalytic BMs with the utilization of sunlight. These functionally engineered structural materials are expected to be utilized for resolving environmental pollutant removal issues in an economic and efficient way. Of course, this area of research is in its infancy. Few reports have demonstrated the potential use of visible light active TiO_2_-inclusive BMs for environmental remediation, and the details are distributed in the forthcoming subheadings dealing with individual cases of pollutant removal. However, marketability, cost of production, and ease of preparation need to be optimized by extensive research in these directions. The ultimate purpose of the incorporation of TiO_2_ into these building materials is to derive photocatalytic properties for the resultant materials. The following paragraphs provide significant research developments that occurred during the years between 2014 and 2019 on TiO_2_-inclusive building materials to achieve the desired NO_x_ removal photocatalytic properties.

## 5. NO_x_ Removal

**Scheme 4:**
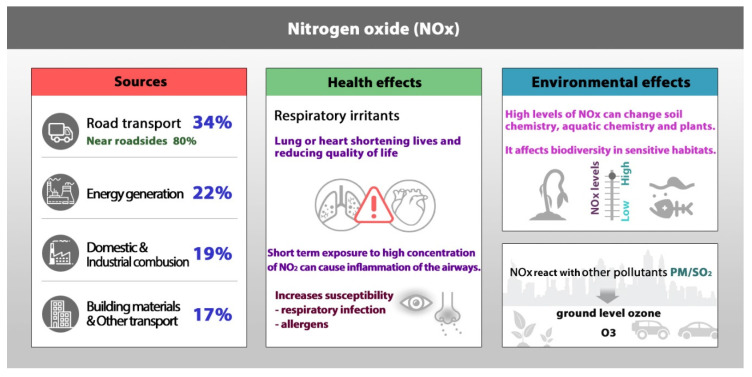
The potential sources and health and environmental effects of NO_x_.

NO_x_ is generally a collective term that represents two distinct gases, namely NO, or nitric oxide, and NO_2,_ or nitrogen dioxide, and the gradual oxidation of NO results in nitric acid and NO_2_. Nitrous acid is a possible intermediate during the transformation of NO to NO_2_. It is to be mentioned that intermediates are more harmful than the primary reactants, NO and NO_2_ [98]. Specifically, NO_2_ is three times more toxic than NO, the primary pollutant [99]. NO_2_ can function as a precursor for the generation of even more toxic atmospheric constituents, such as ozone and peroxyacyl nitrates. In addition, NO_x_ can interact with other primary pollutants, such as PM or SO_x_ (sulfur dioxide and sulfur trioxide), and can generate other problems, such as ozone, acid rain formation, etc. Thus, NO_x_ emission forms the basis of formulating environmental regulations, especially in the ozone non-attainment areas. NO_x_ pollutants are introduced into the ecosystem through natural (extreme heat of lightning), biogenic (fertilization and the use of nitrogen-fixing plants), and industrial (thermal natural combustion, fuel combustion, and prompt process) sources. The probable sources of NO_x_ and the health issues arising from short-term and long-term exposure are presented in Scheme 4. Due to the environmental damage caused by NO_x_ gases, many regulations were designed to limit their emissions. The emission regulations vary from country to country in type and extent. The concentration limit for NO_x_ to cause pollution in ambient air as per the European directive is 40 g·m^−3^ (0.021 ppmv) averaged over one year and 200 g·m^−3^ (0.106 ppmv) averaged over 1 h (Directive 2008/50/EC, 2008) [100]. Likewise, the NO_x_ concentration limits fixed by other authorities, such as WHO, the U.S. Environmental Protection Agency, the Ministry of Environmental Protection of China, and the Australian Department of the Environment and Energy, are similar.

**Scheme 5:**
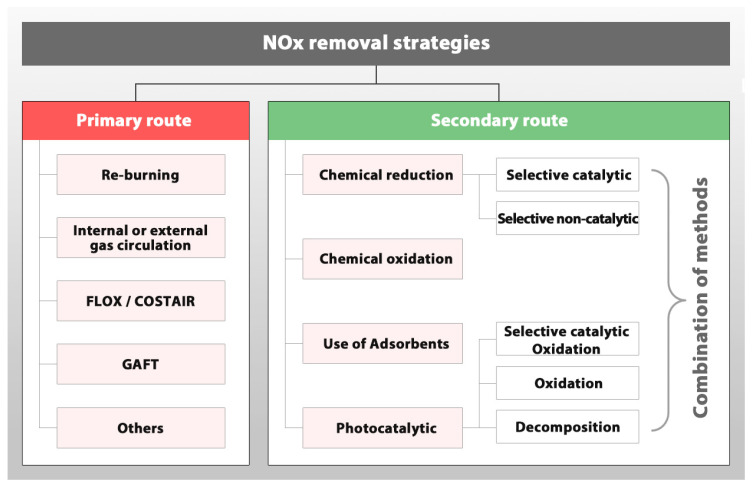
Common strategies (primary and secondary) employed for NO_x_ removal. (FLOX: flameless oxidation; COSTAIR: continuous staged air combustion; GAFT: gas-dynamic abated flame temperature).

Mitigation or removal of NO_x_ can be done through primary and secondary procedures (Scheme 5). Conventional NO_x_ control measures through primary strategies include a selective catalytic reduction (SCR), selective non-catalytic reduction, adsorption, scrubbing (absorption), etc. Traditional systems are constrained by the higher expenses involved, especially for the treatment of sufficiently larger volumes of air comprised of low to moderate concentrations of NO_x_. Besides, conventional systems generate secondary waste, and hence often require further processing. NO_x_ control methodology focuses on either the reduction of NO_x_ back to the parent compound N_2_ or its oxidation to HNO_3_. PCO of NO_x_ offers the following distinctive advantages: (1) non-requirement for extra reactants, and (2) conversion of NO_x_ into nitric acid, a potential raw material for fertilizers. Heterogeneous photocatalysis, which is particularly efficient, includes three different processes: photodecomposition, photooxidation, and photo-selective catalytic reduction. The photocatalytic conversion of NO_x_ to nitric acid comprises a sequential or series of the oxidation process. Higher catalytic activity is ensured by a higher degree of NO and NO_2_ conversions.

NO → HNO_2_ → NO_2_ → HNO_3_

However, the photogenerated charge carriers on the catalyst surface can also stimulate reduction processes. The efficiency of the reduction process of NO_2_ to HONO depends on the reduction capability of the photocatalyst as well [101].

One should note that there is a definite possibility of the generation of NO_x_ in residential (indoor) conditions. Therefore, the quality of indoor air is a matter of concern. It can be noted that an indoor NO_x_ level of more than 200 pg/m^3^ (0.1 ppm) may be reached through NO_x_ generated from unvented gas combustion appliances, and an hourly peak NO_x_ level may be 2000 pg/m^3^ (1 ppm), with a maximum-per-minute peak of 4000 pg/m^3^ (2.1 ppm). In the case of short-term exposure for healthy individuals, NO_x_ concentrations of more than 4700 pg/m^3^ (2.5 ppm), either at rest or with around 2 h of light exercise, can result in pulmonary dysfunction. With regards to long-term exposure (several weeks to months), indoor NO_x_ exposure of nitrogen dioxide with concentrations of less than 1880 pg/m^3^ (1 ppm) can cause a large number of health effects, primarily in the lungs and other organs, such as the spleen and liver. Notably, long-term exposure to NO_x_ can cause reversible and irreversible lung effects. Typically, the impact on the lungs can vary depending on the level of NO_x_ exposure and duration. A low-level NO_x_ exposure of as low as 940 pg/m^3^ (0.5 ppm) may also lead to bacterial infection of the lungs [102].

Few efficient methods have been evolved to minimize the level of indoor NO_x_ emissions inside buildings by utilizing photocatalysis of TiO_2_. The general photocatalytic mechanism involving a sequence of reactions upon irradiation of UV light with TiO_2_ (either pristine or in the form of a building material) is as follows: the exposure of the TiO_2_ surface to a UV light excites the electrons in the valence band (VB) to the conduction band (CB) and, in that process, holes are left in the VB. The generated charge carriers, electrons, and holes subsequently participate in the oxidation-reduction (redox) reactions for NO_x_ degradation. In principle, the generally proposed mechanism of NO_x_ photocatalytic oxidation consists of three stages: reaction between NO and photogenerated OH^·^ radicals which results in HNO_2_ at the surface of TiO_2_ (first stage), transformation of HNO_2_ to HNO_3_ and desorption from the surface of TiO_2_ (second stage), and regeneration of the photocatalyst (TiO_2_) (third stage). The mechanisms involved in these stages of reactions were well detailed by several researchers [100,103]. In this context, a large number of research activities resulted in newly developed/synthesized TiO_2_-based materials that can be applied to buildings in the form of a coating or paint, or mixing with mortar, which will further enhance building aesthetics, sanitation, etc. Considering the advantages of including TiO_2_ into building materials, a flurry of activities has attracted many industrial interests. Several forms of TiO_2_-inclusive building materials, such as concrete pavements, eco-friendly cement (TioCem), and an architectural concrete (white cement), have been evaluated and patented [104,105,106]. Subsequently, photocatalytic paints including TiO_2_ in different kinds of binders, such as lime, polysiloxane, and sol-gel derived silica materials, are most frequently used. The forthcoming headings will describe the importance of TiO_2_-inclusive cement, mortar, concrete, paint, and coatings for NO_x_ removal.

**Figure 1:**
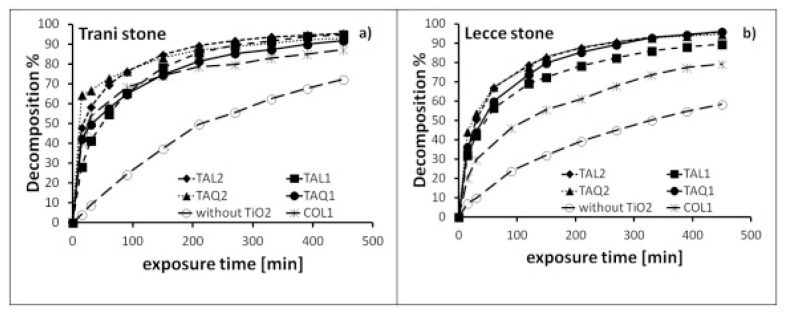
Decomposition rate of Rhodamine B for (**a**) Trani stone and (**b**) Lecce stone as a function of irradiation time [107]. (T: tetrapropylorthotitanate, AL: alcohol, and AQ: aqueous (water). TAL refers to a combination of T and AL in preparation condition, and TAQ refers to the combination of T and water.

Lettieri et al. investigated the influence of colloidal suspensions of TiO_2_ NPs with 1% and 2 wt. % concentrations and evaluated their NO_x_ depolluting abilities by coating them onto the limestone surface [107]. For this purpose, two limestones, namely a compact limestone (PT-Trani stone) and a soft and highly porous limestone (PL-Lecce stone), were utilized. The PL-based surface exhibited higher NO_x_ photo-degradation efficiency than the PT stone-based one under a UV lamp. About 60% of NO_x_ removal was observed over a period of 30 min of exposure over the photocatalytic material coated PL stones (Figure 1). A conclusion was reached with regards to the high NO_x_ removal efficiency derived from TiO_2_-inclusive limestones: the higher surface roughness and porosity of the limestone surface provided an environment for the formation of an extensive exterior surface layer of TiO_2_ NPs. The study also revealed an inadequate reduction of NO_x_ when low porous limestone was used for preparation through alcoholic solutions.

**Figure 2:**
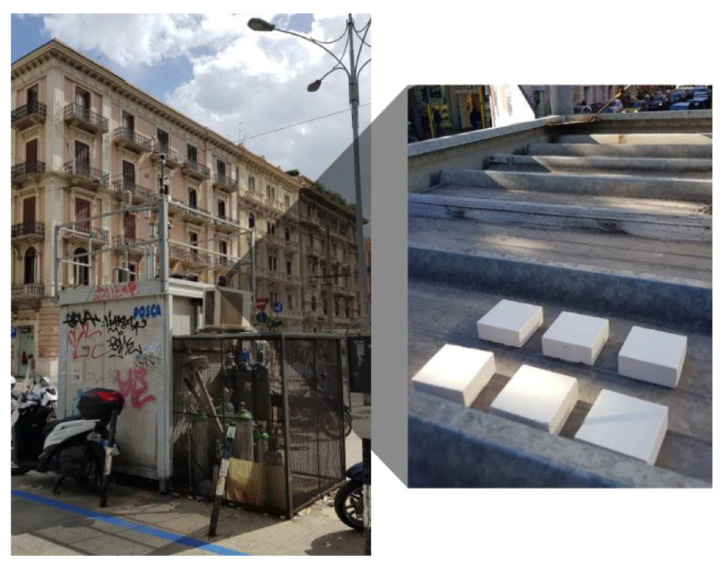
Samples exposed on top of the ARPA (Regional Agency for Environmental Protection) [108].

In a subsequent investigation, the photocatalytic performances of limestone surfaces covered with a coating of water dispersed TiO_2_ NPs were evaluated in an urban environment (Bari, a port city in southeastern Italy from February 2016 to January 2017) (Figure 2) [108]. Data were collected by evaluating the concentrations of air pollutants (PM, NO_x_, CO, and benzene) in urban environmental conditions at the exposure site on a daily average basis for the varying climatic parameters (temperature, rain, wind, and light exposure). The daily mean values for NO_x_ concentrations ranged from 50 to 110 μg/m^3^, with an average of 73 μg/m^3^. The other vital results on NO_x_ removal were as follows: NO_2_ daily mean concentrations were between 30 and 60 μg/m^3^, with an annual average of 45 μg/m^3^. The results were assessed based on the European Union (EU) limit values for NO_2_ concentrations. As per the EU’s suggestion, NO_x_ removal concentrations of annual mean and 18 h mean per year were considered as 40 μg/m^3^ and 200 μg/m^3^, respectively [109].

It was concluded that surface color properties could be preserved after NO_x_ exposure through TiO_2_ coating, and the coating’s effect was lost after eight months with the concomitant decrease in self-cleaning. The utility of a photocatalytic concrete overlay was evaluated in terms of the combined effects of energy conservation in the building, vehicular pollutant removal, and urban heat island (UHI) effect. These effects were compared between gray and white cement, along with a reference to cement without TiO_2_. The environmental benefits of the photocatalytic concrete were inferred in terms of concluding the pavement life cycle assessment (LCA) [109,110].

**Figure 3:**
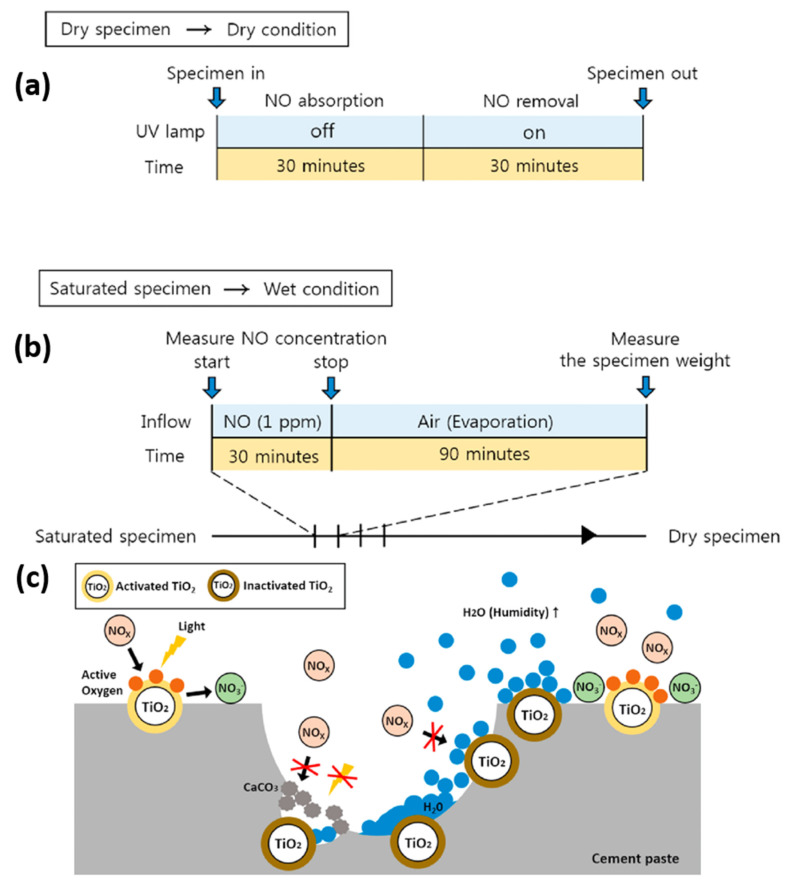
Experimental schemes of (**a**) dry and (**b**) wet condition test and (**c**) photocatalytic cement-based materials (wet conditions) [113].

The TiO_2_-inclusive photocatalytic concrete was effective for NO_x_ removal, which in turn resulted in a decrease in acidification, ecotoxicity, eutrophication, human health degradation factors, smog formation, and respiratory effects. Pollutant removal studies informed that white cement-based photocatalytic materials were superior for NO_x_ removal than gray cement. A microscale thermal model was proposed to explain the UHI effect arising from white cement concrete overlays. The model suggested a five-fold reduction in global warming potential for white cement as compared to gray cement. Through the design of a small scale bed flow photoreactor, the photocatalytic performances of cement-based TiO_2_-containing materials were evaluated towards NO_x_ reduction by monitoring the potential reaction products from the gas phase/aqueous extraction for various experimental conditions (relative humidity, flow rate, mixing ratio, and light intensity) [111]. The results revealed that the potential of the cement-based TiO_2_-containing materials towards photocatalytic remediation of NO_x_ and conversion into nitrite through the analysis of reaction products at the surface. Under atmospheric conditions, the NO and NO_2_ uptake coefficients were determined in the range of 5 × 10^−5^ at a surface deposition velocity of about 0.5 cm/s. The NO_x_ removal capacity of photocatalytic blocks of cement, based on mortars made with Portland cement, two different calcium aluminate types of cement, and air lime, and at least a few TiO_2_ additives (TiO_2_ as reference catalyst, and two doped titania, Fe-TiO_2_, and V-TiO_2_), was investigated [112]. A high NO_x_ abatement (up to 60–80%) with good selectivity was observed for air lime and high alumina cement (HAC) mortar-based photocatalytic cement under the three illumination conditions (UV, solar, and visible light). Lime and HAC mortars exhibited the best NO_x_ abatement percentages under the three irradiation conditions (UV, solar and visible lights). The NO_x_ removal efficiency was explained through the combined influence arising from the presence of higher amounts of calcium carbonate and the probable reaction of the final photo-degradation product of NO_x_, namely nitric acid. The additional role of visible light photo-degradation was proven by the increase in NO removal percentages of the mortars with the doped additives (Fe-TiO_2_ and V-TiO_2_). Among the doped TiO_2_ samples, Fe-TiO_2_ was most effective for NO_x_ removal. Dawa Seo and Tae Sup Yun compared the nitrogen monoxide (NO) removal capacity of TiO_2_-inclusive cementitious materials between wet and dry conditions [113]. An experimental design was made as shown in Figure 3a,b. The wetting of the photocatalytic cement-based materials and the wetting condition play critical roles in achieving a reasonable NO removal rate and also to understand practical sustainability against air pollution. The water molecules were present near TiO_2_ particles, and the wetting altogether influenced the photocatalytic reactions (Figure 3c). The increase in humidity and the rewetting of TiO_2_ occurred through the continuous evaporation of vaporized water molecules from pores.

**Figure 4:**
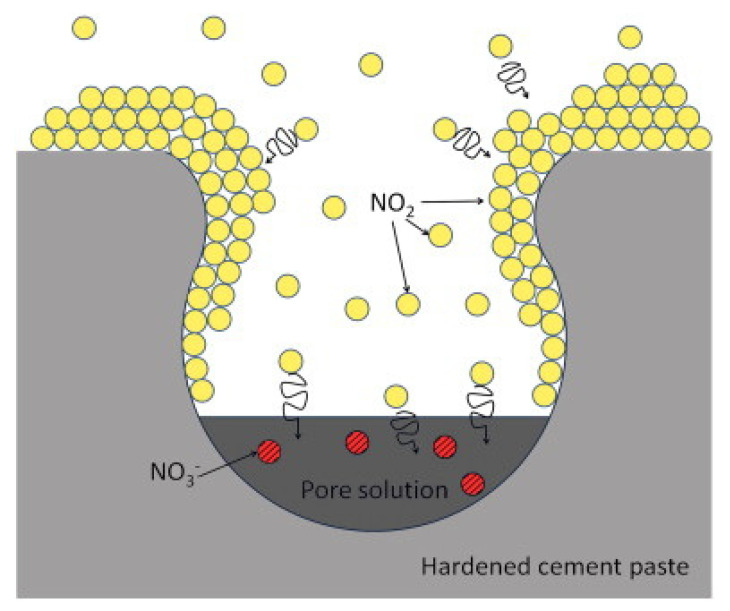
NO_2_ adsorption on hardened cement paste, as well as into the pore solution [115].

Aqueous suspensions of TiO_2_ NP-based photocatalytic surface coatings were developed as a user-friendly approach for cementitious surfaces, and a remediation technique for NO_x_ emission and organic pollutant removal was developed [114]. Hydrophobic and hydrophilic photocatalytic TiO_2_ sols were used for dip coating as well as spray coating on the cement tiles, and NO_x_ removal efficiencies were evaluated. Hydrophobic photoactive TiO_2_ NP-based coatings exhibited an excellent ability to convert NO gas when applied on the concrete surfaces. About a 90% reduction in NO_x_ pollutants was evident from hydrophobic TiO_2_ coated cement tiles for TiO_2_ loadings above 5%, which suggested that the coating can be beneficial for highly polluted areas near pollution sources. The photocatalytic degradation ability for pollutants and the binding efficiency of cement containing TiO_2_ NPs were investigated under nitric oxide and nitrogen dioxide gas exposures in the presence and absence of ultraviolet (UV) light. The photocatalytic efficiency was determined to understand the effect of the different water-to-cement ratios (w/c) of the cementitious materials. Based on the results, a mechanism for photocatalytic NO_x_ removal while using hardened cement was proposed (Figure 4). The mechanism involves the adsorption of NO_2_ gas molecules onto hardened cement paste structures, the occurrence of hydrolysis of NO_2_ into the pores, and the formation of soluble nitrates [115]. These experimental results demonstrated that cementitious materials could be tailored to decrease NO_x_ levels through photocatalysis and showed their ability to bind NO_x_, in particular, NO_2_. The study was designed based on the fact that variation in the water-to-cement ratio can alter the pore structure (i.e., pore volume, size distribution, and interconnectivity) of cementitious materials, which in turn alters the proportion of effective surface area available for PCO and adsorption/binding of by-products.

The TiO_2_-inclusive self-compacting glass mortar (SCGM) was developed for evaluating photocatalytic NO_x_ degradation [116]. Recycled broken glass pieces from a local glass beverage bottle were used as the aggregate. While HNO_3_ is the generally expected final photocatalytic NO_x_ degradation product from cementitious materials, SCGM produces NO_2_^−^ in place of NO_3_^−^ as the major NO_x_ photo-degradation product [117]. The NO_2_ changed to HONO in the atmosphere and caused the generation of secondary pollutants through the photochemical reaction. Based on the use of SCGM-based materials for NO_x_ degradation, it has been inferred that SCGM is not as useful as studies expected for high photocatalytic NO_x_ removal efficiency and high conversion efficiency of NO to NO_3_^−^. Such difficulty in achieving the targeted photo-degradation efficiency demands research on SCGM.

**Figure 5:**
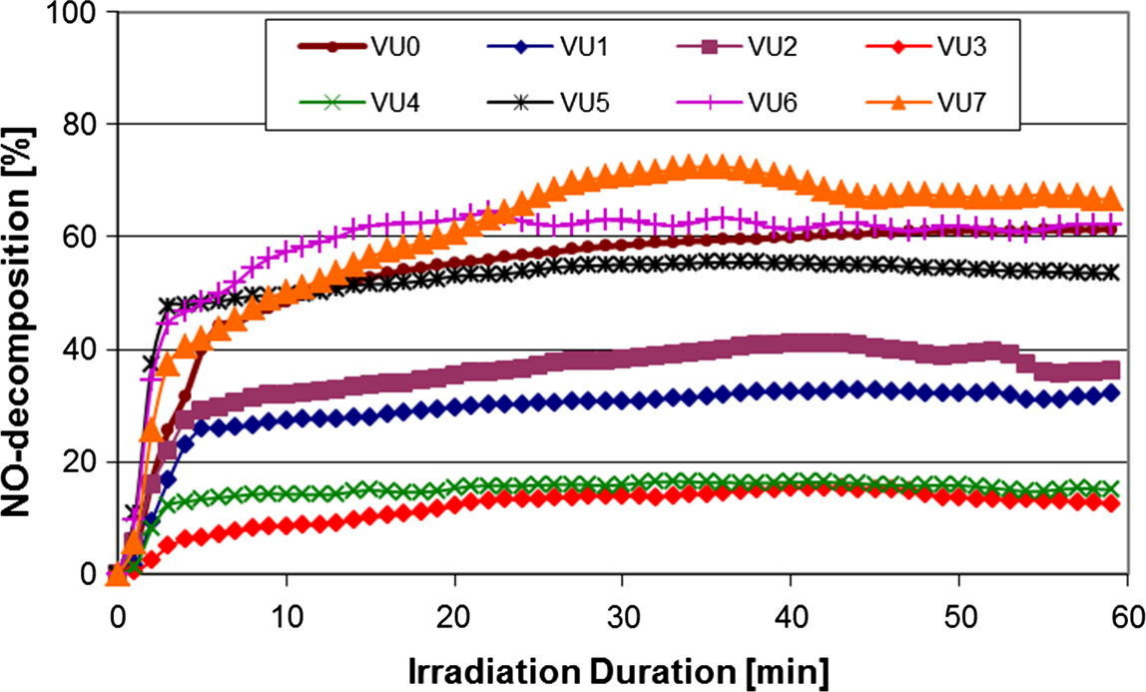
Average degradation efficiencies of NO for all studied specimens [119]. Eight TiO_2_ products (VU0 to VU7) with varying grain size, anatase proportions, and surface area. VU0 is the reference commercial cement mixture.

The on-site determination of NO_x_ photocatalytic removal was performed with eco-blocks fabricated in factories [116]. In another study, specifications according to the Indonesian National Standard were done on determining the composition for the TiO_2_ layer coated over the pavement blocks to have required wear resistance and durability [118]. Filed experiments revealed that the hydrophilic properties of the TiO_2_ surface caused the hindrance of NO_x_ adsorption on the paving block surface because of the competition between NO_x_ and water vapor to reach the active sites of the catalyst. As per the results, 200 g of TiO_2_ per 1 m^2^ square paving blocks was required to transform ambient NO_x_ to nitrate at an average rate of 0.0046 mg/m^2^/min. Other conditions maintained in the study were: average temperature 29.89 °C, humidity 45.31%, and wind speed 0.84 m/s. Functional asphalt pavements were developed by establishing bonding between pulverized TiO_2_-cement mortar and asphalt pavement surface through the epoxy resin. Such a design of functional asphalt showed durable NO_x_ degradation efficiency [119]. The bonded pulverized TiO_2_-cement mortar to asphalt pavement exhibited effective NO transformation, and factors such as the anatase/rutile phase ratio and surface area of TiO_2_ influenced different levels of NO_x_ removal in the range between 14% and 67% (Figure 5).

The NO_x_ degradation capability was evaluated in the test section built at the Institute of Highway Engineering in Aachen, Germany, to understand the applicability of the surface treatment to asphalt pavements. María Pérez-Nicolás et al. modified the mortars with two different binders (Portland cement (PC) and high alumina cement (HAC)) as well incorporating them with nano-structured photocatalytic additives (bare TiO_2_, Fe doped (Fe-TiO_2_) or vanadium doped TiO_2_ (V-TiO_2_) [120]. The inclusion of nano-structured additives resulted in the generation of additional nucleation sites, which in turn caused the acceleration of the hydration process of the cementitious binders. On the other hand, the water absorption was not significant for the photocatalyst-incorporated HAC mortars. As regards to practical application, these systems were beneficial to refine pore structures. Magdalena Janus et al. developed gypsum plates modified by TiO_2_-N, C photocatalysts, and tested for NO_x_ removal efficiency [121]. However, neither the photo-degradation capability towards NO_x_ removal nor the compressive strength were improved through the inclusion of TiO_2_-N, C into the gypsums. The gypsum modified with a higher loading (~20 wt.%) of TiO_2_/N, C, and modified at 300 °C resulted in higher NO_x_ degradation activity. A comprehensive evaluation of the role of TiO_2_ NP content (up to 15%) on the mesoscale (interfacial) and macro-scale (mechanical) properties of the cementitious composites has been brought out through experimental studies [122]. A micromechanical model was proposed to understand the interrelation between meso and macro properties. The proposed model correlated the relation between tensile strain capacity and the ration of the complementary energy to crack tip toughness.

TiO_2_ is used in most paint formulations and finds its application in coating labs and manufacturing sites around the world. The role of TiO_2_ in paints is not only to provide brightness and opacity but also can be extended to NO_x_ control in indoor environments. In principle, photosensitized reactions on TiO_2_ surfaces can influence indoor air quality either beneficially or adversely. For example, NO_x_ may be produced through the reactions of ammonia or deposited nitric acid on the surface of the paint. On the contrary, photocatalytic NO_x_ removal can be beneficially derived through photocatalytic paints. The effectiveness and the outcome of using photocatalytic paints for air pollution abatement purposes is, therefore, a challenging aspect that needs to be adequately addressed to evolve new technologies to reduce indoor NO_x_ emissions.

**Figure 6:**
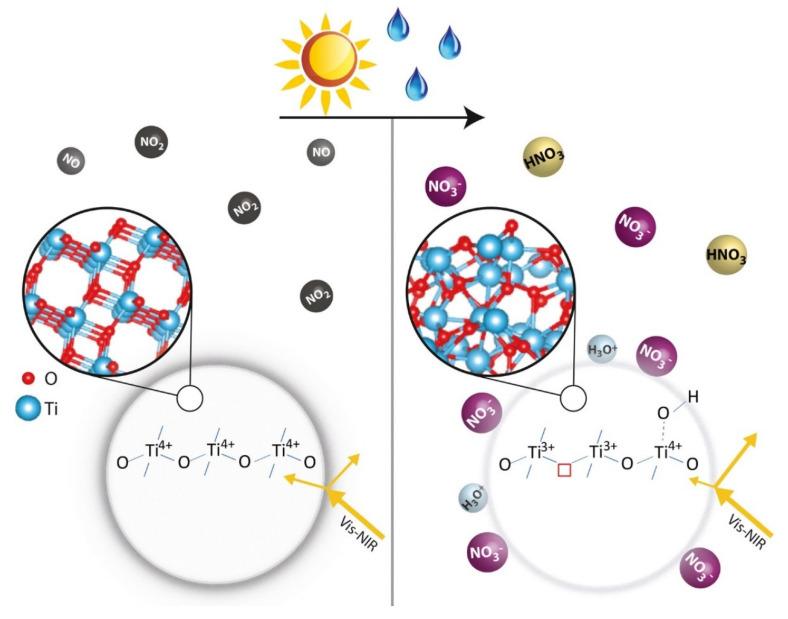
Scheme showing the reactions occurring at the surface of TiO_2_ (anatase phase) [126].

Truffier-Boutry et al. developed macro-sized and nano-sized TiO_2_-inclusive paints and studied their photocatalytic, microstructural, and morphological abilities in the process of understanding their environmental impacts [123]. Their main focus was to understand the main parameters that could control the expulsion of nano-sized particles from nano-TiO_2_ and to bring out safer formulations of paints having salient characteristics, such as less inclusion of nano-sized photocatalytic particles with minimal VOC-releasing possibility. The crucial conclusions derived through the study include that paints formulated with TiO_2_ particles having sizes smaller than 100 nm with rutile crystalline structures exhibited the capacity to remove air pollutants. On the other hand, xylene removal capability was observed for the paint formulation consisting of TiO_2_ NPs. Audrey Bonnefond et al. formulated photocatalytic paints containing two types of polymeric binders [124] (a pure (meth)acrylic latex (LM1), and a fluorinated/(meth)acrylic latex (CS2) with core-shell morphology) along with two types of TiO_2_ NPs (Degussa P25 and Johnson and Matthey (JM) sample) [125]. The substrate film-based paint was superior compared to air film because of the higher availability of interfaces at the bottom of the film for the former paint system. Riccardo Paolini et al. discussed the possible adverse effects of the solar reflectance of TiO_2_-containing materials. An elaborate mechanism to explain the cause for the partial reduction in and decreased crystallinity of TiO_2_ was proposed based on the contact with nitric acid generated by NO_x_ photocatalytic degradation [126]. Upon light irradiation onto the TiO_2_ surface in a humid environment, NOx was converted into NO which in turn hydrolyzed to HNO_3_. The adsorption of nitrates could occur on the protonated TiO_2_ surface arising from the acidic environment, causing an increase in oxide defect density and a decrease in crystallinity, or an increase in amorphous characteristics. The proposed mechanism (Figure 6) presents explanations for the variation of the optical behavior of TiO_2_ in terms of changes in morphology, surface area increase, surface defects, chemical structural changes (hydroxylation, protonation), adsorption, etc. [127].

**Figure 7:**
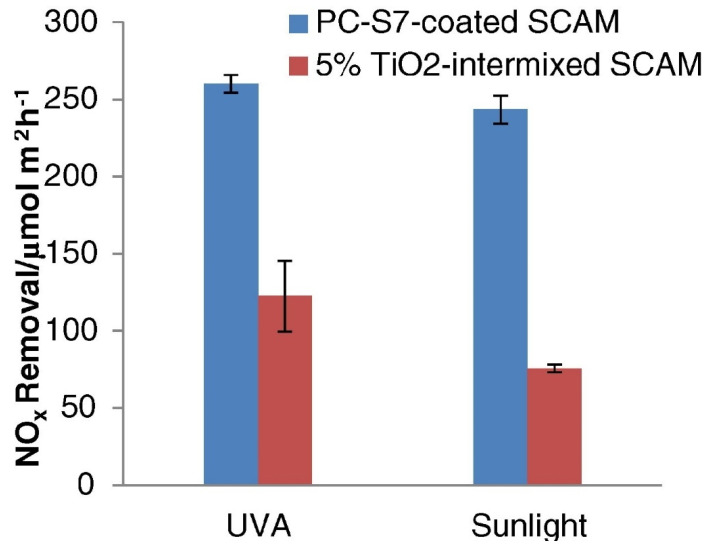
Photocatalytic NO_x_ removal on architectural mortar samples (PC-S7-coated and 5% TiO_2_-intermixed) under UV-A and sunlight irradiation conditions [128].

They concluded that the study could be useful to evolve strategies for developing newer self-cleaning TiO_2_-based construction materials with enhanced optical properties. In the process of developing functional cement-based photocatalytic products, Ming-Zhi Guo et al. formulated a modified self-compacting architectural mortars (SCAM) by applying a TiO_2_ containing paint as the surface coating [128]. The photocatalytic NO_x_ removal performance was compared between the SCAM samples prepared through brush coating of TiO_2_ containing paint (brush coated) and a simple non-paint formulation containing 5% TiO_2_ (P25) (Figure 7) [128]. Two kinds of irradiations were used. Higher NO_x_ removal efficiency was noticed for the PC-S7-coated SCAM sample coated with paint than the 5% TiO_2_-intermixed samples under both UV-A and sunlight irradiations. The bonding between the TiO_2_ particles and the SCAM surface was more robust for the brush coated SCAM than the dip-coated ones. They concluded that the TiO_2_-coated SCAM products can be used as exterior finishing materials of buildings in urban environments.

**Figure 8:**
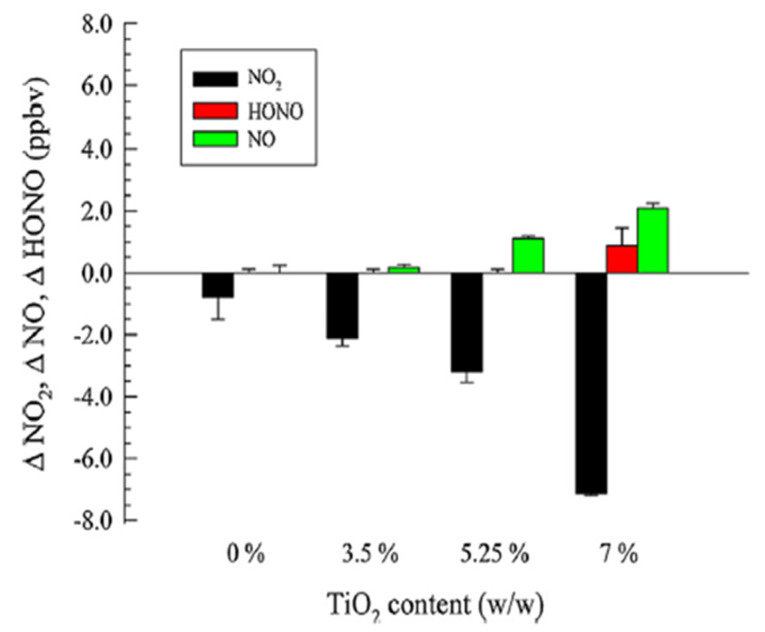
Formation of NO_2_, NO, and HONO on photocatalytic paints with 0%, 3.5%, 5.25%, and 7% of TiO_2_ NPs (NO_2_ mixing ratio of 40 ppb and RH of 40%) [129].

Adrien Gandolfo et al. developed different quantities of TiO_2_ NP-inclusive photocatalytic paints and investigated the optimization of conditions for NO_x_ remediation and HONO formation. The photoactivity of the developed paints was tested under a UV light in an indoor condition. The results revealed that the photocatalytic paints substantially decreased the NO_2_ concentration [129]. The photocatalytic paints including 0.3% to 7% TiO_2_ NPs were studied using a horizontal flow tube reactor. The NO_2_ uptake showed an increasing trend concerning TiO_2_ loading for the photocatalytic paint with a loading of up to 7% TiO_2_ NPs. Notably, the photocatalytic paint loaded with 7% TiO_2_ efficiently eliminated the majority of gas-phase NO_2_, converting it to NO and HONO (Figure 8). Notably, the NO conversion rate increased with the increase in TiO_2_ NP content. Gas-phase HONO was predominantly formed for paint including 7% TiO_2_ NPs.

**Figure 9:**
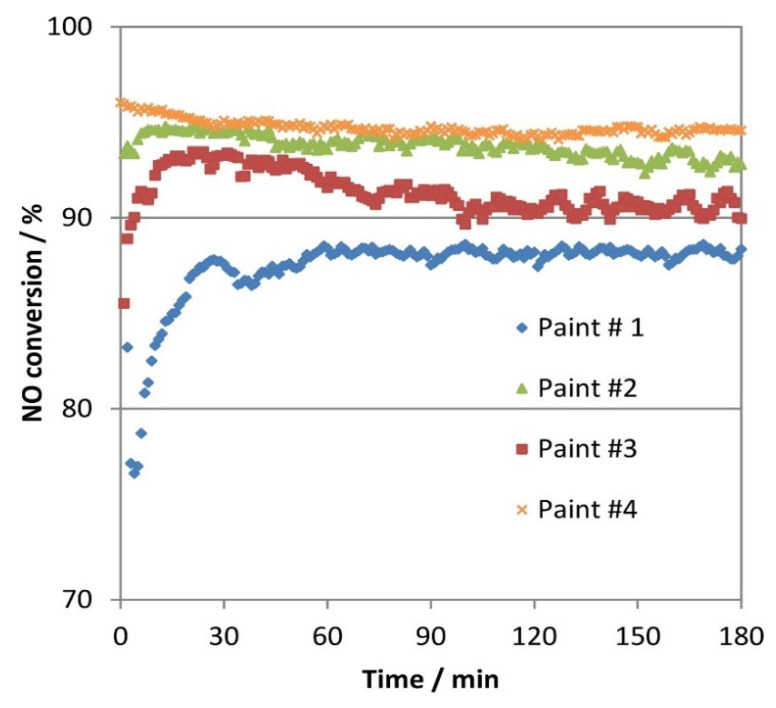
NO conversion for the four tested paints [131]. Paint #1 to Paint #4: Paints formulated with varying compositions of TiO_2_, CaCO_3_, P25, and PC500.

A dynamic mass-balance model was proposed to predict the time-based formation of HONO in a real indoor condition. The photo-efficiency of four kinds of TiO_2_ coatings along with the influence of a SiO_2_ interlayer was investigated on NO_x_ photo-degradation [130]. Three types of commercial TiO_2_ suspensions and a home-made Titania sol-gel were used, and a SiO_2_ layer was formed on the cement surface. The coating of the SiO_2_ layer in between the mortar and the commercial TiO_2_ coatings could not stabilize the TiO_2_ NPs. On the other hand, the homemade TiO_2_ NPs were stabilized through excellent adhesion arising from the probable interactions between SiO_2_ and TiO_2_ sols. Joana Ângelo et al. evaluated outdoor NO_x_ abatement using photocatalytic paints incorporated with commercial TiO_2_ (Figure 9) [131]. Commercial samples of P25 from Evonik and PC500 from Cristal were utilized for developing exterior water-based paints. The standard ISO 22197-1:2007 protocol was used to evaluate the photocatalytic NO_x_ degradation performance of these four paints. Upon performing outdoor tests, the developed paints showed a higher NO conversion rate (more than 80%). To quote correctly, the paint comprised of PC500 and calcium carbonate exhibited the highest NO conversion of ~95%.

Luna and coworkers optimized the preparation of Au-TiO_2_/SiO_2_ coatings and demonstrated the effectiveness of the photocatalyst compositions towards photooxidation of NO, adopting a protocol (ISO 22197-1) for monitoring NO in air purification [132]. A three-stage methodology—(i) preparation of Au dispersion, (ii) mixing with commercial TiO_2,_ and (iii) integration of Au-TiO_2_ with silica—was adopted. The observed selectivity for NO_x_ removal has been attributed to the enhancement of photoactivity by Au NPs as well as the possible activation of O_2_ and NO molecules through strong adsorption that could augment NO oxidation. Besides, the photoactivity of Au-TiO_2_/SiO_2_ under visible light has been demonstrated for other polluting substances, such as dyes and 4-nitrophenol.

Following the success of the preparation of the Au-TiO_2_/SiO_2_ photocatalyst, Luna and coworkers extended their research on the production of Au/N-TiO_2_/SiO_2_ photocatalysts [133]. A modified procedure was adopted that included urea as the nitrogen source. NO abatement tests using the Au/N-TiO_2_/SiO_2_ under UV light and the selectivity towards NO removal were demonstrated. Under similar Au loading, the Au/N-TiO_2_/SiO_2_ photocatalysts showed more photoactivity than other reference catalysts (without nitrogen doping in TiO_2_). Further to the demonstration of the photoactivity of Au/N-TiO_2_/SiO_2_ for NO removal, experiments ware extended on the photocatalyst coating performances by selecting three different building materials (Capri limestone, Grey Pearl granite, and HERPLAC^®^ concrete) [134]. The coatings were formed by spraying onto one of the larger faces until its saturation. Factors such as porosity in the substrates, the TiO_2_/SiO_2_ ratio of the coating, the amount and size of Au particles, and nitrogen doping could be tuned to achieve good photoactivity.

## 6. Volatile Organic Compounds

VOCs are one of the four organic compounds classified as very quickly volatile or semi-volatile organic compounds included with particulate (especially organic) volatile organic materials [135]. In another version, the VOCs can be referred in terms of boiling points (between 50 and 260 °C) for highly reactive and/or toxic organic compounds. The exposure to VOCs can cause health issues, such as respiratory diseases (eyes/nose/throat irritations), neural disorders, and sick generating syndrome (non-specific symptoms and ill-feeling health). VOCs can also be the reason for the formation of secondary pollutants, such as ozone. VOCs can cause concern both in the form of indoor and outdoor air pollutants. Indoor environments can be polluted with VOCs because of the usage of building materials, paints, fuels, cleaning materials, pesticides, materials, etc. Indoor VOC concentrations are generally 5 to 10 times highrt than outdoor environments, which, in turn, depends on the climate and other parameters [136]. Nearly fifteen years ago, an analysis performed on the existence of VOCs within a sufficiently aerated residence (without the usage of carpets and drapes) revealed the presence of the VOCs and, for easy reference, were listed by descending concentration (selected ones are given here): toluene, benzaldehyde, mono(2-ethylhexyl) phthalate, decanal, p-xylene, 1-butoxy-2-propanol, 2-butoxyethanol, undecane, β-pinene, decane, o-xylene, tetradecane, acetic acid, pentadecane, dodecane, menthol, ethylbenzene, styrene, tridecane, benzyl alcohol, camphene, methyl salicylate, naphthalene, madendrene, etc. [137]. A review published in the year 2009 details the significant changes in building materials and indoor commercial environments over the past half-century and explains the origin of some of the of VOCs in indoor environments [138]. Another review was published in the year 2016 that briefly details the general photocatalytic oxidation approaches that have been applied for the removal of VOCs and formaldehyde in indoor environments [139]. The review also provides details on potential sources for indoor expulsion of VOCs arising from building materials (Table 1).

The list of indoor VOCs is expected to include several more additional volatile organics in recent years because of modernization and unlimited opportunities for the use of household materials. These details and the possible concern arising from the potential exposure to VOCs in indoor air have triggered the development of new home construction techniques/materials to improve indoor air quality significantly.

### TiO_2_ Based Photocatalytic Composites for VOC Removal

TiO_2_-inclusive photocatalytic materials were developed to degrade indoor VOC pollutants under UV light irradiation [140,141]. Several TiO_2_-based photocatalytic materials have been developed for the decomposition of VOCs [142,143,144,145]. These reviews, published in the years between 2014 and 2019, focus the discussion on the utility of TiO_2_-based photocatalytic building materials towards VOC abatements. The report also summarizes the development of TiO_2_-based (doped, composite, nanostructured, etc.) photocatalytic materials that have been tested for VOC removal capability (Table 2) but still need to be tested through inclusion into building materials. The utilization of the reported TiO_2_ materials for building material development will form the platform for exploring the possibility of the development of advanced building material fabrications in the years to come.

**Figure 10:**
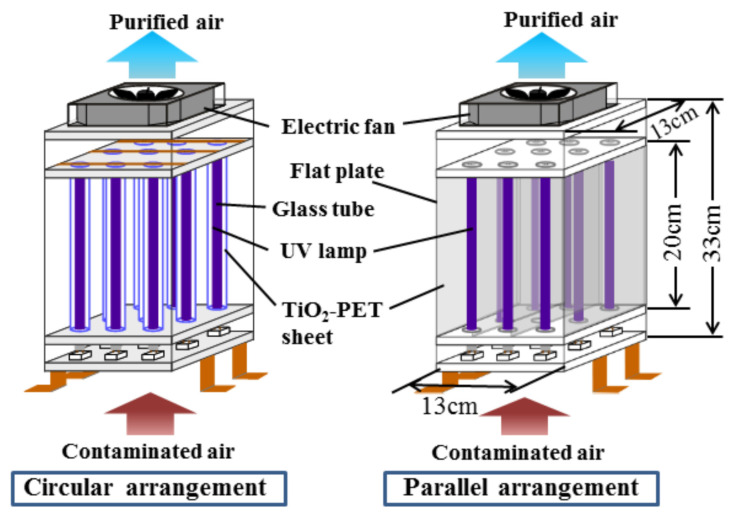
Arrangement of UV lamps and TiO_2_-PET sheets (surface area of the TiO_2_-PET sheet) [174].

**Figure 11:**
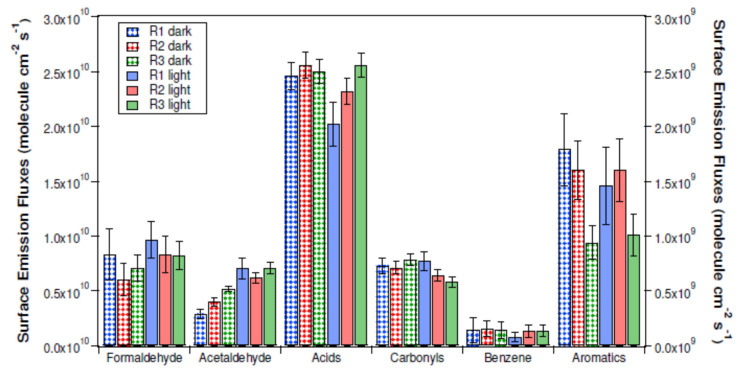
Surface emission fluxes (molecule cm^−2^ s^−1^) obtained for selected VOCs (formaldehyde, acetaldehyde, acids, carbonyls, aromatics, and benzene) under “dark” conditions (lozenge) and in the presence of a UV light (solid) [175].

Fumihide et al. compared the efficiencies of the photocatalytic decomposition of VOCs between two arrangements, namely, parallel and circular, for the removal studies (Figure 10). The parallel arrangement was designed by keeping the UV lamps parallel to flat TiO_2_-PET sheets. In the case of a circular arrangement, a UV lamp was held in a glass tube, and the cylindrical TiO_2_-PET sheet was arranged close to the surface of the lamp [174]. The photo decompositions of acetone, isopropanol (IPA), and toluene were studied. The circular arrangement outperformed the parallel one and was preferred for optimal VOC decomposition. A general conclusion was drawn based on the results. The complete decomposition of VOCs, including their intermediates, could be achieved with a UV intensity above a certain threshold and the choice was also based on the types of VOCs present. Julien Morin et al. used a horizontal flow tube reactor to study VOC emissions from paints that included mineral silicate binders, acrylic binders, and acrylic/siloxane binders with/without incorporated TiO_2_ NPs [175]. In the majority of the experiments, it was found that the mineral binder-inclusive paints emitted fewer VOCs as compared to the acrylic binder-inclusive ones. The surface-emission fluxes of the VOCs, such as formaldehyde, acetaldehyde, benzene, aromatics, acids, and carbonyls, were studied under UV irradiation for the photocatalytic paints. The results of mineral silicate and acrylic/siloxane binders were compared with an acrylic binder to infer the impacts and effects of the paint composition. The surface-emission fluxes of carbonyl compounds were presented and discussed (Figure 11). They concluded that the binder inclusion did not significantly enhance the properties of the photocatalytic paints concerning VOC emissions. Nejc Rozman et al. prepared rare earth element (such as La, Gd, and Ce) modified TiO_2_ and assessed the photocatalytic degradation of a few VOCs [162].

**Figure 12:**
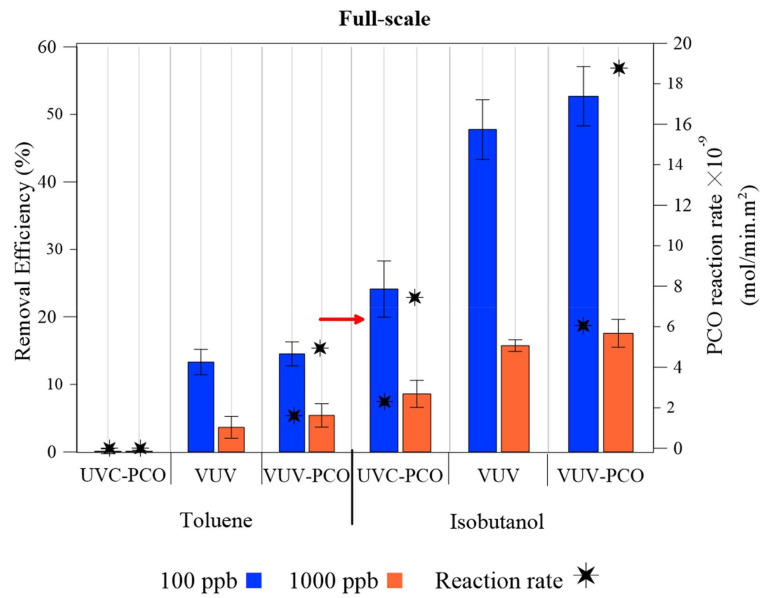
Removal efficiency of toluene and isobutanol and PCO reaction rate for different systems (UVC-PCO, VUV photolysis, and VUV-PCO) (V = 1.25 m/s) [177]. PCO: photocatalytic oxidation; UVC: ultravacuum, VUV: vacuum ultraviolet.

A reactor comprising of a gas pump and pipes was set up to understand the degradation kinetics using Fourier-transform infrared spectroscopy. The research paper reported comparative data on the photocatalytic isopropanol oxidation between simulated solar light and UV irradiation as well as pristine TiO_2_ and rare earth element-doped TiO_2_. Birte Mull et al. developed pure TiO_2_ or TiO_2_ modified with graphene oxide (GO)-inclusive photocatalysts and investigated the photocatalytic degradation of a few of VOCs (butyl acetate, toluene, and limonene) under UV LED (light emitting diodes)/blue LED light irradiation conditions [176]. Based on irradiation experiments, they inferred that the modified TiO_2_ samples with trace amounts of GO (0.75–5%) showed better photocatalytic performance under blue light. However, photocatalysts loaded with a higher value of GO (10% or higher) caused adverse photoactivity both under UV and blue light irradiation conditions. Zahra Shayegan et al. investigated the three levels (bench-scale, pilot, and full) of scaling effects on the removal efficiency of toluene and isobutanol removal efficiency as well as the generated by-product by evaluating the UV-irradiated photocatalytic oxidation performance using a commercial TiO_2_ photocatalyst (Figure 12) [177]. Based on the factor θ (mg VOCs/g photocatalyst media), a comparison between the three scales (full, pilot, and bench) was made on the gravimetric (per mass) pollutant removal efficiency for toluene and isobutanol in terms of the flow rate of pollutants and the weight of the photocatalyst. Conditions such as residence time, flow rate, and humidity were optimized to derive extrapolated information for real indoor application. Full-scale studies showed that toluene could not be effectively removed in UV-assisted PCO due to the higher velocity of flowing gas and lower light distribution. However, studies performed with 100 ppb and 1000 ppb concentrations of isobutanol showed a 24.1% and 8.6% removal efficiency, respectively (Figure 12).

**Figure 13:**
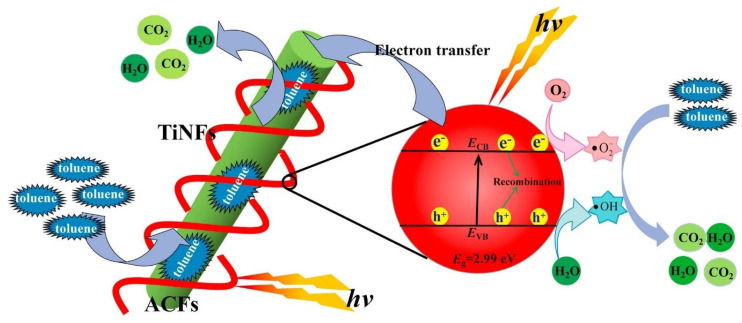
Scheme depicting the adsorption and photo-degradation synergetic model of TiO_2_ nanofibers/Activated carbon fiber (TiNF/ACF) porous composites towards toluene removal [178].

Alireza Haghighat Mamaghani et al. presented a comprehensive overview of commercial TiO_2_ photocatalyst-based VOC removal in air [14]. Enhanced photocatalytic activity could be achieved through fine-tuning physical properties (crystallinity, crystal size, anatase and rutile phase compositions, and porosity) and surface properties (amount of surface hydroxyl groups, surface area, and surface density) of the TiO_2_. The role of support materials, such as activated carbon, on the adsorption and removal efficiency of VOC was detailed. Ming-Jun Tian et al. prepared a porous photocatalyst composite comprised of ultralong TiO_2_ nanofibers (TiNF) distributed in activated carbon fiber (ACF) (TiNF/ACF) porous composites by the depositing TiNFs within ACF felts, the support matrix [178]. They observed improved photocatalytic performance for toluene removal because of synergetic effects arising from the interaction between the TiNFs and ACFs (Figure 13). A mechanism for efficient photocatalytic activity was suggested. ACFs play multiple roles, such as hindering the recombination of electron–hole pairs, enhancing the light adsorption ability by reducing the TiO_2_ bandgap energy (Eg) to 2.99 eV, and increasing the quantum efficiency of TiO_2_. Typically, a high surface area and macroporous structure of composite photocatalysts could result in higher adsorption efficiencies (98.9%) for a toluene concentration of <4600 ppm. Zhuowei Cheng et al. used a combined sol-gel hydrothermal methodology for the preparation of La/N-TiO_2_ nanotubes [179] and evaluated photocatalytic activity towards chlorobenzene. A greater surface area and smaller particle sizes were produced with the co-doped TiO_2_ nanotubes. The relative humidity of the system played a role in the conversion of chlorobenzene. A probable mechanism involving a water molecule for the photocatalytic chlorobenzene conversion is presented.

D. Truffier-Boutry et al. formulated paints based on micro-sized and nano-sized TiO_2_ particles and studied their VOC emission/removal efficiency [123]. However, the results informed that few of the organic compounds, with a predominance of the harmful pollutant formaldehyde, and other compounds such as methanol, acetaldehyde, were removed upon light irradiation. D. Enea et al. evaluated the photocatalytic activity of three photocatalytic paints prepared with different TiO_2_ samples under UV as well as solar light irradiation, considering the degradation of 2-propanol as the probe molecule [180]. The results informed that the photocatalytic activity of the paints was influenced by the changes in the surface hydrophilicity and the aging process. Natan Blommaerts et al. evaluated the gas-phase degradation of acetaldehyde by photocatalytic paints coated over the entire interior parts of the spiral tubes and assessed the gas-phase photocatalytic degradation of acetaldehyde [181]. Superior photocatalytic performances were noticed for both the single and double cylinder coated cylinders over a vast set of experimental conditions. They derived reasons for the superior VOC degradation efficiency of the spiral reactors over the conventional photocatalytic reactors. The possible reasons could be because of (1) the probability of uniform illumination over the entire reactor length, (2) optimized light transmission, and (3) effective contact establishment between the gaseous reagents and the irradiated surface over the whole tube length. A comparative result informed that a spiral reactor could show a 100% acetaldehyde degradation efficiency for a low residence time of 60 s. On the other hand, 100% acetaldehyde degradation could be achieved from the annular reactor even at 1000 s residence time.

Wenda Zhu et al. developed photocatalytic coatings for barn walls and ceilings for mitigating indoor odors based on a few of the new TiO_2_-samples, PureTi Clean, Evonik, and P25 [182]. They utilized these paints to understand their effects in decreasing odorous VOC emissions through stimulated evaluating, with stimulated conditions having few organic compounds like diethyl sulphide, dimethyl disulfide, dimethyl trisulfide, butyric acid, p-cresol, and guaiacol. The pure Ti-based coating, with a loading of 10 μg/cm^2^ photocatalyst, showed the highest removal rate for a few of the selected odor compounds.

C. Toro et al. studied the uptake coefficients of a few of the selected VOCs onto commercial TiO_2_ coating in a continuously stirring reactor tank [183]. The molar yield and the composition of the aldehyde mixture were monitored during the removal of each VOC. The highest level of acetaldehyde production was evident from toluene photooxidation, while the lowest was from mesitylene. The Ag^+^ doped TiO_2_ was synthesized and successfully incorporated into a natural hydraulic lime mortar [184]. The gas-phase VOC photo-degradation capability of the modified lime mortar was assessed under the irradiation of artificial visible light. The 1 wt.% and 5 wt.% Ag^+^ doped TiO_2_ incorporated mortar samples produced 1.8 ppm/h and 2.9 ppm/h of acetone, respectively. A non-linear relationship between photocatalytic activity and wt.% Ag^+^ TiO_2_ was noticed. The modified mortar exhibited VOC degradation under artificial visible light of the kind used for indoor illumination, without any UV component. The photocatalytic activity towards VOCs was lower than that of NO_x_ abatement. A mechanism for the visible light photoactivity was presented. The Ag NPs clustered nearer to TiO_2_ NPs could harvest visible light and trigger the PCO of VOC gases, specifically causing the degradation of isopropanol into acetone. Magdalena Janus et al. prepared several concrete-TiO_2_ or C-TiO_2_ composites using N-TiO_2_ or C-TiO_2_ and concrete as raw materials [185]. The modification of TiO_2_ with N or C enhanced the photocatalytic oxidation process for gaseous pollutants.

## 7. Self-Cleaning

Ever since the concept of “self-cleaning” was initially unveiled based on the superhydrophobic property of lotus leaves, the importance of the self-cleaning principle has been realized towards developing many innovative superhydrophobic materials by researchers from surface chemistry to material science utilizing them for practical applications. The three processes, namely wetting, dewetting, and adhesion of the surface, are, in turn, related to self-cleaning and can be understood using contact angle (CA) measurements. CA refers to the angle between the liquid-solid interface and the liquid-vapor interface. The assessment of CA measurements can be performed in terms of advancing angle θ_A_, static contact angle θ, receding angle θ_R_, and sliding angle α [186].

Based on the CA, the nature of a surface is defined as hydrophilic when θ_R_ is <90° and hydrophobic when θ_R_ is >90°. Additionally, a superhydrophobic surface with θ_A_ is ≥145° does not have an affinity for water. Superhydrophobic properties arise for non-smooth/rough surfaces having ≥145° and α as <5–10° with possible hysteresis in the CA. The superhydrophobic property of lotus correlates with θ > 150° and a small α < 2°. Interestingly, other unusual characteristics, such as superhydrophobic super-wetting, are related to surfaces having a strong affinity for water and liquid surfaces. The term “superhydrophilicity” was first introduced by Fujishima et al. [187] and subsequently, a flurry of research activities were triggered on superhydrophobic properties of surfaces and their associated applications [188]. Dreich and Chibowski redefined superhydrophobic materials as rough and porous materials having a surface roughness (Wenzel) factor larger than 1. On superhydrophilic surfaces, water (liquid) spreading occurs entirely [189]. In general, the removal or cleaning of surface-coated/adhered contaminants from the surface is dependent on surface properties, such as wettability and surface energy, which in turn decide the mobility of water on the surface. The wettability of the surface is dependent on the morphology, free energy, and chemical properties of the surface, along with the viscosity and other properties of the spreading liquid [190]. It must be noted that effective surface cleaning by water for both superhydrophobic and super hydrophilic surfaces can be explained by various mechanisms [191,192,193]. Different models are available to explain the surface-wetting process over multiple surfaces [194,195,196,197,198].

In 1997, the self-cleaning and antifogging characteristics of the TiO_2_ surface under UV light irradiation were first demonstrated by Fujishima et al., and the results revealed that superamphiphilic surfaces were produced through photo-initiated processes [194]. The irradiation of anatase TiO_2_ coated on a glass substrate generated photocatalysis and photo-induced super hydrophilicity, simultaneously, through intrinsically different methods. Typically, the surface of anatase TiO_2_ coated on a glass substrate, which was having a water-contact angle of 72° ± 1° before UV irradiation, changed to a surface that spreads water after UV irradiation. This breakthrough research initiated a new era on TiO_2_-based self-cleaning materials. Extensive research activities on TiO_2_-based self-cleaning materials opened up application avenues for these materials in different disciplines, such as environment, energy, and industries, and generated a scope for new business/markets.

In recent years, TiO_2_-based photocatalytic materials have been effectively utilized for mitigating environmental pollution and damage control on a global scale, leading to the development of commercial products [191,199,200,201,202,203]. Further, the development of TiO_2_-based advanced materials for viable environmental cleanup has attracted a great deal of attention. Now, a variety of TiO_2_-based materials have been commercialized, arising from their unique photo-induced properties. TiO_2_-based self-cleaning materials can destroy bacteria that adhere to its surface under UV or visible irradiation. The superhydrophilicity property of self-cleaning TiO_2_-based materials favors the complete spreading of water droplets at a faster speed on their surface which contributes to the removal of contaminants/pollutants adhered on the surface. With regards to construction materials, coating materials, such as glass, tiles, fabrics, cement, and paints, were developed utilizing the photo-induced hydrophilic conversion of TiO_2_ surfaces. In the past few years, self-cleaning materials have attracted much attention, and a series of TiO_2_-based self-cleaning coatings have been fabricated on a variety of substrates [204,205].

Studies informed that the augmented self-cleaning property of TiO_2_ could be achieved when the surface was exposed to a stream of water flow. Therefore, self-cleaning can be effectively implemented with TiO_2_ coatings on the exterior of buildings because the exterior walls are expected to be exposed to abundant sunlight and natural rainfall. A variety of TiO_2_-based self-cleaning construction materials, including cement, tiles, plastics, glass, aluminum sliding, etc., have been commercialized. On the practical side, glass surfaces coated with TiO_2_ NPs and having self-cleaning properties can be seen at the National Opera Hall, China [191]. At the Dives in Misericordia Church, Rome, Italy, self-cleaning white cement consisting of TiO_2_ NPs has been utilized as the construction material [84]. In Japan, a more significant number of buildings have been constructed with self-cleaning tiles including TiO_2_. The PanaHome Company from Japan popularizes “eco-life” type houses constructed with self-cleaning windows, solar cell-inclusive roof-tops, and tiles [206].

Strategies were evolved to enhance the efficiency of the photocatalytic and self-cleaning properties of TiO_2_ through doping with metals/non-metals, generation heterojunctions between TiO_2_ and other low bandgap semiconductors, and the inclusion of graphene. The enhancement in the photoactivity of TiO_2_ was explained in terms of bandgap tuning, creation of localized energy levels within the bandgap, and generation of intrinsic defect sites. Several reviews have been published on the self-cleaning action of TiO_2_ over the past years which mainly discuss the photocatalytic activity of TiO_2_ [39,205,207]. In recent years (2015–2019), researchers developed self-cleaning building materials based on cement, concrete, or asphalt concrete, with the inclusion of surface-modified, functional component-incorporated, or doped TiO_2_. These advanced building materials endowed buildings with desirable functionalities which ensured the improvement in construction quality. The details of the type of building materials that were developed using pristine/physically/chemically modified TiO_2_, the methodologies adopted, the tests that enabled the self-cleaning property, and the salient results of the published papers on the topic are tabulated for reference (Table 3). A few of the studies not listed in Table 3 are detailed as below.

An Au-TiO_2_/SiO_2_ coating was applied by a simple spraying method on two different building stones in the process of developing a self-cleaning BM [208]. The self-cleaning and depolluting properties were evaluated using soot and NO, respectively. The results informed that the highest photoactivity level was observed for coating formulations having smaller sized Au NPs (~13 nm) and intermediate gold loading (0.5% w/w Au/TiO_2_). The self-cleaning and photocatalytic properties of a TiO_2_/AuNRs-SiO_2_ coating on fossiliferous limestone (calcite 98.5%, α-quartz 1.5%) were evaluated under a solar light irradiation condition with reference to MB discoloration and carbon soot removal [209]. An interesting observation in this work was the effect of calcination temperature on the self-cleaning and soot removal characteristics. A calcination temperature of 450 °C provided the best self-cleaning properties which was attributed to the increased contact between the Au and SiO_2_ particles.

In another work, effective and long-term Au-TiO_2_/SiO_2_ coatings for BMs were developed by dispersing Au and TiO_2_ NPs into an initial sol containing a silica oligomer and *n*-octylamine [210]. The surfactant used in the formulation had multiple roles such as: to catalyze the sol-gel transition of the silica precursor, to generate a mesoporous silica gel network, and to increase the contact of contaminants to photoactive centers. An enhanced TiO_2_ photoactivity level was observed under solar radiation, even for the inclusion of a smaller amount of Au NPs into the self-cleaning coating, because of the absorption of a larger proportion of visible light (~45%).

## 8. Issues and Limitations

The achievement of long-term usage and high efficiency in PBMs are important goals for the commercial/practical utilization of PBMs. However, these goals are challenging to realize because of the inherent limitations of pristine TiO_2_ (visible light inactivity, high charge recombination rates, etc.) as well as the modifications in the properties of TiO_2_ (partial deactivation or blocking the active sites by intermediates or products) while PBMs are subjected for use in building environments. A few of the important issues that need to be resolved towards achieving high efficiency include (1) decreasing e^−^-h^+^ recombination rates, (2) increasing active sites on the surface of TiO_2_, (3) maximizing the dispersion of TiO_2_ within the matrix of the BM (cement, mortar, etc.), (4) optimization of cluster size and porosity, (5) ensuring of maximal photocatalytic activity for both large dyes and small gaseous molecules, (6) introduction of TiO_2_ in the form of an active mixed composition of its polymorphs (e.g., keeping suitable proportions of anatase and rutile phases), and (7) improving the pore structure of cement through control of the hydration process. Other factors that need to be considered include light sensitization of TiO_2_ by the other components present in the PBM, geographical applicability of PBMs (i.e., the influence of latitude, seasons, and time of the day on their photocatalytic performances), selectivity towards pollutant removal, the influence of photocatalytic reaction by-products, the adverse health impacts of the by-products, the surface chemistry of the particles, the durability of TiO_2_ in BMs, and dispersibility of TiO_2_ in cementitious materials.

The effects of TiO_2_ NPs on the inherent properties of hardened cement pastes and mortars are still to be thoroughly examined as the literature provided controversial information on the question of whether the addition of TiO_2_ introduces pozzolanic activity, or whether TiO_2_ acts simply as a non-reactive filler with photoactivities. A study which describes the preparation of 4% TiO_2_ cement revealed the acceleration of calcium silicate hydrate (C-S-H) gel formation at the early stage of hydration and the consequent formation of crystalline calcium hydroxide [241]. The enhanced strength and improvements in the microstructure of the TiO_2_-added cement were observed [241]. These researchers came out with a conclusion that TiO_2_ acted simply as a non-reactive fine filler and did not generate any pozzolanic activity. However, TiO_2_ functioned as a nucleation site for the accumulation of hydration products. Further, the influence of TiO_2_ NPs on the mechanical properties of cement-based composites is not clear. Generally, it is understood from the literature that the addition of nano-TiO_2_ particles decreased the setting time and improved the strength of mortars at early stages. On the other hand, the influence on the long-term mechanical properties of cured samples is disputable.

The various organic and/or inorganic compounds that are added while making PBMs influence their photocatalytic efficiency. It has been notified that the included inorganic or organic compounds within BMs degrade at varying rates, causing changes in the exposure of TiO_2_ to have light activation. Research indicated that stable refractory oxides and mineral acids present in BMs could bind to the catalyst surface and block catalytic sites [242]. The intermediates formed during the photocatalytic process could block the photoactivity of TiO_2_. Deactivation of TiO_2_ is a crucial disadvantage for the oxidation of VOCs or organic pollutants [243]. The poisoning effects of partially oxidized intermediates are believed to be responsible for this deactivation. A study informed that photocatalytic oxidation of the formation of partially oxidized intermediates (e.g., benzaldehyde and benzoic acid) during the photooxidation of toluene caused deactivation by blocking the activation sites [244]. Deactivation during ethanol and acetone PCO has also been reported [245,246,247]. These studies demonstrated the loss of photocatalytic activity as a result of oxidation of hydrocarbons, and suggested that partially oxidized intermediates are the reason for reduced photoactivity. Nevertheless, the encouraging fact is that these intermediates undergo slow photooxidation and, as a consequence, photoactivity can be restored upon continued exposure to oxidizing conditions. It should be noted that the high photocatalytic activity of TiO_2_ under UV light irradiation can also induce the inevitable degradation of surrounding materials, which ultimately causes the loss of appearance and severely decreases the lifetime of the paint [248]. For example, the unwanted photocatalytic reactions can either promote polymerization of the organic binder, creating cross-linking to result in embrittlement [249] or decompose the binder into VOC components. This could affect the surface of the building in the form of surface roughening and chalking. Besides, the photocatalytic degradation of pigments could cause discoloration [250].

TiO_2_-based nanocoatings could be applied to the external walls of buildings for their antibacterial and self-cleaning properties [251,252]. These photocatalytic coatings, when present on external surfaces in real-life conditions, are subjected to various mechanical solicitations and environmental weathering [253,254]. As a consequence, there could be a consequent loss of their structural integrity, resulting in the degradation of the BM. Furthermore, the disintegration of the structural material could result in the release of NPs into the gaseous or solution environments. It has been noted that TiO_2_ could convert atmospheric ammonia into toxic nitrogen oxides, which in turn contribute to the proliferation of urban smog [255]. In the process of broadening the practical applications of PBMs, a few promising strategies to improve the specific surface area of the photocatalysts have been attempted [256]. Studies have focused on creating a new class of nano-admixtures in cement-based composites. In this regard, the combination of nano-silica and TiO_2_ particles has been tried in order to synergistically derive the beneficial properties of both of them when included in cement-based composites [229,257,258]. Extensive research activities are demanded in these directions with economically viable materials.

## 9. Conclusions and Future Perspectives

The development of advanced building materials offers the opportunity to incorporate additional novel functionalities (such as self-cleaning, pollutant removal capability, durability in fluctuating climate, etc.) and provides a platform for addressing new challenges. These advanced building materials are judiciously integrated with new technologies in both residential and commercial buildings, in the process of developing smarter, more energy-efficient, and more secure infrastructures that will address environmental and global social challenges. Advanced building materials are utilized not only for resolving problems associated with indoor air pollution in buildings but also for mitigating gaseous emissions exceeding the allowable concentration in the atmosphere from traffic in the building’s surrounding area. In this regard, scientific developments in modern material synthesis and nanotechnology provide an adequate platform for the design and creation of new and smart building materials with tailored properties.

Photocatalytic materials can degrade air pollutants and can also clean surfaces and act as a photoactive paint, mortar, etc. Concretes made with these types of materials are given primary importance for their applications in road infrastructures, such as lining and coatings for tunnels, and also accessories such as barriers and walls. A massive surge of research and commercial activities has prompted the production of building materials including photocatalytic TiO_2_ particles. In particular, TiO_2_ demonstrated its prominence for the development of photocatalytic building materials due to its advantageous characteristics that include ease of manufacturing or synthesis, non-toxic nature, less corrosive property, excellent chemical stability, low cost, and inherent photocatalytic properties. In general, the photoactive TiO_2_ in building materials absorbs ultraviolet rays and oxidizes most organic and some inorganic pollutants. The vast number of research activities that are being continuously conducted by researchers forms the basis for building engineers to design modern strategies towards conceiving advanced building materials. Portland blocks of cement containing TiO_2_ were developed and used in the form of pavements, concrete blocks, roof tiles, etc. Notably, the photoactive cement-coated walls removed air pollutants that can discolor exposed surfaces and added additional built-in functionalities, such as a self-cleaning property that supports the cleaning of residual products by rain or by the flow of water. TiO_2_-inclusive photoactive cement/binders meet many objectives, such as sustainability, self-cleaning of the surface coupled with redox reactions induced by sunlight or ultraviolet light, photo-induced hydrophilicity of the catalyst surface, mitigation of the urban heat island effect arising from the photocatalytic properties of these “cool” materials. Paved surfaces (e.g., highways, runways, parking lots, etc.) generally account for up to ~60% of developed urban areas, and could be modified with a thin layer of TiO_2_-inclusive photocatalytic materials so that the topmost surface effectively clears the environment of the adverse effects of pollutants coupled through the influence of solar reflectance. In addition, a simple renovation of the top surface of pavements can regenerate their photoactive surface. Urban temperatures could be controlled by simply coating wall surfaces with white photocatalytic materials. Such applications can be extended to parking lots and roads to reduce temperatures and to cool the environment, as well as to improve air quality. Additionally, with the correlation between the formation of smog and the relative warmth of the environment, white photocatalytic coatings can also contribute to a reduction in ozone formation during the summer period. This effect will, in turn, result in the decrease in the kinetics rate for photochemical pollution reactions, and will consequently decrease the formation of secondary harmful substances. At a close glance at the market for TiO_2_-based photocatalytic building materials, a large number of companies (>2000) have developed building materials in the form of exterior construction materials (coated glass, paints, tiles, and tents), interior decoration products (antibacterial and self-cleaning tiles, smart windows, wallpapers, etc.), air purification systems (air cleaners, air conditioners, air cleaner hybrids, etc.), and water purification facilities (waste and sewage water purification). However, these developments have been made amidst several issues of using TiO_2_ as a photocatalyst, despite its various beneficial aspects.

One of the significant issues with TiO_2_ photocatalysis is the restricted visible light photoactivity for TiO_2_ due to the large bandgaps (3.0–3.2 eV) of traditional TiO_2_ materials. Together with the fact that TiO_2_ photoactivity is mainly centered on only the ultraviolet (UV) spectrum (~3% of the solar irradiation received on Earth), there exists a great challenge in expanding the photoactivity of TiO_2_ into the visible region. Besides, the problem associated with the rapid spontaneous combination of photon-excited electrons and holes for traditional TiO_2_ materials should be circumvented for enhancing photoconversion efficiency under direct solar light. Thus, there is a continuous need for developing TiO_2_ photocatalysts with good charge separation, increased surface reactions, and extended optical absorption to visible light. Approaches that have been attempted towards achieving visible light active TiO_2_ include the modification of TiO_2_ via metal-ion implantation with transition metals, doping with metal or non-metal, compositing TiO_2_ with low bandgap semiconductor, TiO_2_ dye-sensitization, doping with luminescence materials and metal-nonmetal co-doping, etc. (Scheme 3). While the majority of the research activities on TiO_2_ modification for visible light activity have been reported in terms of the inclusion of the modifying precursor during sol-gel synthesis, limited studies were available on post-modification after TiO_2_ synthesis. Moreover, many of the modification approaches are time-consuming, constrained with multiple steps, and involve expensive modifier(s), which ultimately excludes the possibility of commercial building material production. Hence, research nees to focus on developing economically viable TiO_2_ modification procedures, especially for building material applications.

This review details the strategies that have been adopted for achieving high photocatalytic performance and visible light photoactivity for TiO_2_ in the last few years (2014–2019), including controlling the geometric shape or morphology of the particles, particle size, and porosity tuning, bandgap alteration, surface defects or disorder manipulations, phase composition control, etc., through chemical, physical, and mechanochemical approaches. Research activities focusing on enhancing photoactivity and improving visible light photoactivity, additional aspects, such as adsorption characteristics and surface hydrophilicity, and photocatalytic kinetics have not been adequately considered. Hence, further research activities are needed on these areas that have been neglected. Based on the basic and fundamental research activities, several advancements have been achieved regarding the use of TiO_2_ as a photocatalyst; in particular, improvements in photocatalytic efficiency, expansion of optical absorption to visible regions (solar light), and more straightforward chemical/physical modifications were made over the last few years. We believe that the knowledge gathered through the literature on these developments will form the basis for the development of advanced next-generation building materials. Besides, one can conceive more socially relevant applications for TiO_2_; as a part of its application in aspects of photocatalytic building materials, there needs to be extensive activity on the renovation of tunnels with advanced photocatalytic technology, as this would be beneficial in terms of the actual costs involved for refurbishment with paint and UV lighting systems. This application can also resolve the pollution problems of those who are residing in the vicinity. The photocatalytic cleaning of these types of activities would serve as a cheaper means of protecting the surroundings. This comprehensive review will hopefully be useful for scientists and engineers having the intention to translate basic research information into practical and commercial products for construction purposes.

## Figures and Tables

**Table 1:** Possible sources of indoor pollutants of volatile organic compounds (VOCs).

VOCs	Potential Sources
Formaldehyde	Pesticides, flooring/insulating/wood materials, coatings, paints, etc.
Acetaldehyde	Flooring/wood materials, etc.
Naphthalene	Insulating/mixed materials, etc.
Chloroform	Glues, pesticides, etc.
Ethylbenzene	Adhesive, paints, furniture, etc.
Dichlorobenzene	Wood/ceiling materials, etc.
Carbon tetrachloride	Paints, coatings, industrial cleaning agents, etc.
Toluene	Pesticides, flooring/insulating/wood materials, adhesives, paintings, etc.
Others (ketones, esters)	Perfumes, paints, adhesives, plastics, resins, disinfectants, etc.

**Table 2:** Summary of TiO_2_ based photocatalytic composites for NO_x_ removal.

Modification Type	Specific Example	Methodology	Remarks on NO_x_ Removal	Reference
**Composites**	Vanadium/TiO_2_	Functional theory calculations combined with in-situ diffuse reflectance infrared Fourier transform	Selective photooxidation-stable formation of V/TiO_2_ surface	[146]
	P25 (titania) functionalized with thioglycolic acid (TGA)-capped CdTe colloidal quantum dots (QDs)	Chemical methods	TGA, CdTe, and titania components in the TGA-CdTe/P25 composite	[147]
	Hydroxyapatite (HAp)/beta-tricalcium phosphate/TiO_2_	Intermixing and a post-curing coating procedure	Active sites are available on the surface of the material leading to greater NO_x_ oxidation	[148]
	Composites of TiO_2_ (Hombikat, P25, sol-gel synthesis) and zeolite ZSM-5	Sol-gel and solid-state dispersion	Well-dispersed TiO_2_ particles over the ZSM-5 crystals caused good NO_x_ removal	[149]
	TiO_2_ covered with a thin layer of an alumina–silica	Commercial	Coating TiO_2_ with Al_2_O_3_ and SiO_2_ dramatically increases the conversion of NO_2_ to NO on TiO_2_	[150]
	TiO_2_ photocatalyst with Ba species	Impregnation	Ba–Ti mixed oxide phase was formed-the surface of amorphous Ba–Ti mixed oxides could store NO_3_^−^ more densely than the TiO_2_ surface	[151]
	TiO_2_–SiO_2_ mixed oxides	Blast furnace, Chemical method, and calcination	High activity in selective catalytic reduction, excellent stability, and low preparation catalyst cost	[152]
	TiO_2_ and ZnO	Plasma treatment	Longer reaction times for efficient oxidation of NO_x_	[153]
	Fe/TiO_2_	Co-precipitation	Fe increased the electron-hole separation efficiency because Fe^3+^ incorporated into TiO_2_ can act as an electron-trapped agent	[154]
	Au/CeO_2_^−^TiO_2_	Chemical method	Au^−^Ce^3+^ interface formed and served as an anchoring site of the O_2_ molecule. Then more adsorbed oxygen could react with photogenerated electrons on TiO_2_ surfaces to produce more superoxide radicals for NO oxidation	[155]
	TiO_2_/Al_2_O_3_binary oxide	Sol-gel synthesis protocols	Alumina domains can be utilized as active NO_x_ capturing sites that can significantly eliminate the release of toxic NO	[156]
	TiO_2_-Carbon	Chemical method	Reduction of NO towards N_2_ by proton adsorption-promotion on Pt-Pt sites or by selective-secondary sites (as titanium dioxide)	[157]
	TiO_2_/G hybrids	Sol-gel	Interaction between TiO_2_ nanoparticles and graphene sheets, acting as electron traps.	[158]
	G included pure rutile TiO_2_ nanowire	Electrospinning, sol-gel, and calcination	The inclusion of G into TiO_2_ nanostructures enhances the visible light photoactivity	[33]
	TiO_2_/G	Solvothermal process	Favorable NO_2_ adsorption on graphene through an interaction between the NO_2_ molecules and the conjugated system in graphene sheets	[159]
	TiO_2_/Printex U (a famous model carbon black)	Coating	Reduction of NO to nitrogen, achieving DeNO_x_ without any reducing agent	[160]
**Doping**	Ti^3+^ self-doped TiO_2_ NPs	Spray-coating	Surface oxygen vacancies caused by the diffusion of photo-generated holes play significant roles-Ti^4+^ is reduced to Ti^3+^, dissociating water molecules and facilitating the adsorption of −OH species on surfaces-photoinduced hydrophilicity	[161]
	Modified with Ce, La, and Gd	Chemical method (gel precipitation) and hydrothermal treatment	The addition of rare earth elements to hydrothermal processing slowed the anatase-to-rutile and brookite-to-rutile transformation-grain boundary pinning effect	[162]
	TiO_2_ plates doped with Ba, La	Calcination	Doped with 5%-Zr^4+^ and calcined at 800 °C, resulted in 100% degradation of NO_x_ as compared to commercial P25. The addition of Zr^4+^ caused a decrease in crystalline sizes of both anatase and rutile forms causing an increase in surface area	[163]
	Ni doping	Thermal treatment	6% weight Nickel doped KA100 and annealed at 1000 °C showed excellent NO_x_ removal. Transformation into 100% rutile phase and size effects played roles in photoactivities.	[164]
	Cu, Zn, and Cu-Zn	Thermal treatment	0.25 Cu/0.75 Zn-Ti450 (Cu:Zn molar ratio equal to 1:3) was the best performing specimen in degrading NO_x_	[165]
	Carbon-doped titanium dioxide and nano-silica	Coating and UV-pre treatment	Highest NO degradation is achieved by the two-layered coatings comprised of carbon-doped TiO_2_ and silica	[166]
	Nitrogen modified TiO_2_	Sol-gel green synthetic method	Heated at 450 °C possessed excellent photocatalytic activity under visible white-light (indoor artificial lighting)-double PCA than P25 TiO_2_ NPs.	[167]
	Barium-modified titanium dioxide	Impregnation method	BaO species, which was generated from the decomposition of Ba(NO_3_) _2_, works as a NO_x_ storage material	[168]
**Nanostructuring and physical modifications**	The electronic crystallographic structural relationship for Ti_0.909_W_0.091_O_2_N_x_	Mixing and calcination	Oxygen vacancy acts as an electron trapping center for conduction-band electrons and reduction of NO	[169]
	TiO_2_ to detect secondary aerosol pollutant formation	Seed via atomization	Reactive carbonyl compounds caused by the photo-degradation role of TiO_2_ lead to the suppression of C formation	[170]
	Titanium dioxide nanosheets and anatase titanium dioxide nanospindles with highly exposed (001) facets	Hydrothermal and faceted engineering	Preferential exposure of (001) facets of TiO_2_ had a positive effect on increasing the specific surface area of the catalysts	[171]
	Size controlled nanoparticles	Controlled annealing	Mortar mixtures with only 1 wt.% TiO_2_ resulted in NOx degradation rates close to 80%	[172]
	Crystalline mixed-phase (Anatase/Rutile) mesoporous	Calcination at temperatures (600–800 °C, 1 °C/min) followed by a second heat treatment	A synergetic effect between anatase and rutile	[173]

**Table 3:** Summary of various TiO_2_ based pristine/modified building materials having self-cleaning properties, their preparation route, and salient results.

Type of Building Material	Pristine/ Modified TiO_2_	Preparation Method	Testing Mode	Salient Results	Ref.
Cement paste	Pristine TiO_2_	Mixing with cement paste (0.5~7.5% by weight of cement)	Photo-degradation of methylene blue (MB) under UV light	Photo-degradation ratio by 9.5% (0.5% for the plain)	[211]
Cement paste	Modified TiO_2_	Sol-gel dip-coating on cement paste surface	Photo-degradation of methyl orange (MO) under UV light	MO degradation efficiency by 90% in 2 h	[212]
Cement paste	Modified TiO_2_	Mixing with cement paste (1%, 5% and 10% by weight of cement)	RR198 dye degradation under UV light	RR198 dye degradation rate by 60%	[213]
Cement paste	Pristine TiO_2_	Mixed with cement paste (5% and 30% by weight of cement)	Congo Red (CR) dye degradation under a UV lamp and visible lamp	Absorbance decreases by 94% in 5 h using P25	[214]
Cement paste	Pristine TiO_2_	Mixed with cement paste	Photo-degradation of MB under UV light	Photo-degradation ratio by 100% in 3 h	[215]
Cement paste	Pristine TiO_2_	Coating on a cement paste substrate	Photo-degradation of MB under UV light	Performance similar to the reference material P25 in 2.5 h	[216]
Cement paste	Pristine TiO_2_	Mixed with cement paste (2% by weight of cement)	Photo-degradation of Rhodamine B (RhB) under UV irradiation	Photo-degradation rate by 90% in 60 min	[217]
Cement paste	Modified TiO_2_	Mixed with clinker	Photo-degradation of MB under UV light at the age of 6 months	Photo-degradation rate by 25% in 4 h	[218]
Cement paste	Modified TiO_2_	Spraying TiO_2_ sols on cement paste	Photo-degradation of RhB under UV irradiation	Photo-degradation rate by 65% in 9 h	[219]
Mortar	Pristine TiO_2_	Mixed with cement (0.5~10% by weight of cement)	Reflectance recovery subject to artificial UV or direct sunlight irradiation	Reflectance recovery by 85% in 8 h under UV irradiation	[220]
Mortar	Pristine TiO_2_	Mixed with cement (2% by weight of cement)	Discoloration of organic dyes (RhB and MB) under UV irradiation	Color change by 40~60% in 24 h under UV irradiation	[221]
Mortar	Pristine TiO_2_	Mixed with Mortar	Monitoring the variations in color under sunlight	After aging, photoactive samples showed limited color variation	[222]
Mortar	Pristine TiO_2_	Mixed with Mortar (2.5~10%)	Photo-degradation of Rh Bunder daylight lamp	RhB dye degradation rate by 85% in 24 h	[223]
Mortar	Pristine TiO_2_	Coating TiO_2_ dispersed in ethanol on the mortar surface.	Photo-degradation of soot concentration under UV light	Photo-degradation ratio by 70%	[224]
Mortar	Pristine TiO_2_	TiO_2_ intermixing and coating	Photo-degradation of Rh Bunder visible light	RhB removal over 50%	[225]
Mortar	Modified TiO_2_	Spraying TiO_2_ suspension on mortar	Photo-degradation of RhB under UV irradiation	RhB removal by 80% in 3.5 h	[226]
Mortar	Pristine TiO_2_	Mixed with mortar (1~5% by weight of a mixture)	Photo-degradation of diesel exhaust soot under UV light and sunlight	Slightly higher degradation of organic matter on the surface for the mortar with higher TiO_2_ content	[227]
Mortar	Pristine TiO_2_	Coated on mortar substrate and mixed with mortar	Photo-degradation of RhB under UV irradiation	RhB removal by 65% after weathering	[228]
Mortar	Modified TiO_2_	Mixed with mortar (3% by weight of mortar)	Photo-degradation of RhB under mercury lamp	RhB removal by 100% within 1 h	[229]
Mortar	Pristine TiO_2_	Mixed with mortar (2.5% and 5% by weight of mortar)	Photo-degradation of soot concentration under UV light	Promotion of self-cleaning effect for TiO_2_-containing mortars after UVA irradiation.	[230]
Mortar	Pristine TiO_2_	Use of ready-mixed mortar containing TiO_2_	Photo-degradation of RhB under UV irradiation	RhB discoloration rate by 55% in 26 h	[231]
Mortar	Pristine TiO_2_	Spraying TiO_2_ sols on mortar surface	Photo-degradation of Rh Bunder fluorescent lamp	RhB photo-degradation rate by 100% in 5 days	[130]
Mortar	Pristine TiO_2_	Coating on mortar surface	Photo-degradation of RhB under UV irradiation	RhB removal by 65% in 26 h	[128]
Mortar	Modified TiO_2_	Mixed with mortar (3% by weight of cement)	Photo-degradation of MB under UV light	Photo-degradation rate by 35% in 4 h	[232]
Mortar	Pristine TiO_2_	Mixed with mortar (2% by weight of cement)	Nitroblue tetrazolium (NBT) ink color change under UV light	NBT testing takes substantially less time (10 min) than conventional photocatalytic activity tests	[233]
Mortar	Modified TiO_2_	Dip-coating of TiO_2_/SiO_2_ sol on mortar surface	Photo-degradation of RhB under UV irradiation	Photo-degradation rate by 90% in 5 h	[234]
Concrete	Modified TiO_2_	Spraying TiO_2_ suspension on a porous concrete	Photo-degradation of MO under UV light	Photo-degradation ratio by 35% in 3 h	[235]
Concrete	Pristine TiO_2_	Slurry method (SM) Cement mixed method (CMM) Epoxy sealer method (ESM) Waterproof sealer method (WSM)	Phenol removal efficiency under UV light	Phenol removal ratio by 85% in 4 h (ESM)	[236]
Concrete	Pristine TiO_2_ and Modified TiO_2_	Spray and dip coating	Photo-degradation of MB under UV light	MB degradation by 5.2 × 10^−4^ pixels^−1^	[114]
Concrete	Pristine TiO_2_	Spraying TiO_2_ suspension on concrete	Photo-degradation of MO under UV light	MO degradation efficiency by 100% in 2 h	[237]
Concrete	Modified TiO_2_	Coating on a concrete substrate	Photo-degradation of MB under UV light	Photo-degradation ratio by 100% in 5 h	[238]
Concrete	Pristine TiO_2_	Mixed with concrete (3.5% by weight of cement)	Photo-degradation of MB under UV light	Photo-degradation ratio by 82% in 26 h	[239]
Concrete	Modified TiO_2_	Spraying TiO_2_ sols on the concrete surface	Photo-degradation of Rh Bunder natural condition	RhB photo-degradation rate by 30% in 24 h (for the specimen at the age of 2000 h)	[240]
